# Numerical study on existing RC circular section members under unequal impact collision

**DOI:** 10.1038/s41598-022-19144-1

**Published:** 2022-08-30

**Authors:** Liu Yanhui, Khalil Al-Bukhaiti, Zhao Shichun, Hussein Abas, Xu Nan, Yang Lang, Yan Xing Yu, Han Daguang

**Affiliations:** 1 School of Civil Engineering, Southwest Jiaotong University, Chengdu, Sichuan China; 2 Department of Civil Engineering and Energy Technology, Faculty of Technology, Art and Design, Oslo Metropolitan University, Oslo, Norway

**Keywords:** Civil engineering, Mechanical properties

## Abstract

Traffic accidents and derailed train-related incidents have occurred more often than ever in recent years, resulting in some economic damage and casualties. Reinforced concrete (RC) constructions often involve derailed train and vehicle accidents. Rarely are such side collisions studied in previous studies. To do this, high-fidelity simulation-based finite-element (FE) models are created in this paper to accurately simulate the collision of circular RC members with a derailed train. The reinforced concrete member structure is common in high-speed railway stations. The impact energy of the impact body is significant, causing structural member failure. It analyses the dynamic behavior of reinforced concrete members under unequal span impact loads. Numerical implementations of impact issues are discussed from the perspective of geometric, contact, and material properties. The reliability and precision of the ABAQUS code to solve impact issues are verified by comparing failure modes, impact, and deflection time history experimental outputs. By analysing the impact response characteristics, used the control variables to study the failure process and mode (including the characteristics of impact and reaction forces, deflection time history curve, impact force–deflection curve, and bearing reaction force–deflection curve). The reinforcement ratio, impact velocity, concrete strength, and slenderness ratio significantly affect shear crack pattern and development. Changes in impact velocity and slenderness ratio also affect member failure modes.

## Introduction

Reinforced concrete structures are susceptible to impact in normal use or natural disasters, such as the impact of various vehicles on the piers of urban overpasses and pedestrian bridges, the impact on indoor parking lot columns, the impact of ships on bridge piers, dock infrastructure, and derailed train on metro station building as well. These impacts sometimes not only lead to local damage to the structure but may even cause the collapse of the entire building, resulting in incalculable casualties and economic losses. The reinforced concrete structures will frequently be subjected to sudden loads such as impacts, earthquakes, and explosions during service life.

The strength, deformation, elasticity, and confining effect are influenced due to the changes in the cross-section, reinforcement, and the width-to-thickness ratio of the members under the lateral impact loads^1–4^. There has been some investigation into these factors in the previous literature. The effects of the geometric shape parameters (circular, hexagonal, rectangular, and square sections)^5–9^ on the material properties of the hollow steel tubes and CFST specimens subjected to axial compression tests were studied^10–16^. The results showed that the circular specimens are the ideal samples from axial stress and ductility values.

Hu et al.^17^ studied the confining effects of concrete-filled steel tubular columns under axial compression due to the change of section shape. The circular steel tube has a greater confinement effect on the concrete than the square section. It is less prone to local buckling, especially when the width-to-thickness ratio of the cross-section is relatively small. It can be found that hollow reinforced concrete columns with the same cross-sectional area are less prone to torsion than solid reinforced concrete columns because of their relatively large torsional stiffness. The structural stability can be effectively improved when subjected to external loads^18^.

Meanwhile, special-shaped reinforced concrete columns could usually meet the requirements of building functions. The failure modes of reinforced concrete members under lateral impact are quite different. The failure mode of reinforced concrete beams gradually changes from bending failure to shear failure as the impact velocity increases^19–21^, especially severe diagonal cracks generated and punching shear failures will be formed at the impact location in the middle of the specimen with a short time when suffering high-speed impact^22,23^.

While the reinforced concrete members usually produce large deflection deformation at the impact point with unequal impact load position, and the flexural crack occurs with the steel rebar^1–3,24,25^.

Also, another study examines how effective shear reinforcement is as a rehabilitation strategy for PT "post-tensioned" slabs that falling boulders have injured. After the accident, rebuilt both slabs by replacing damaged components and installing shear ties to avoid future collapse.

The impact test was performed following the repairs, and both punched shear capacity and normal stresses were measured^26^. The explosion in Beirut on August 4, 2020, is regarded as a case study in a structural engineering technique using nonlinear computational finite element modeling of the silos.

This study aims to assess the structural response of grain silos to enormous blast loads. The explosion's size is defined as the magnitude of the computer model that causes the same silo damage and swaying seen on-site. In addition, the damage to the standing silos has been evaluated, and concluding suggestions have been provided by Yehya Temsah et al.^27^. In the past, experimental, analytical, and numerical studies have been performed to determine the RC columns' behavior under the impact loads caused by vehicle collisions. The constitutive relationship (material level) under dynamic material load is important for numerical analysis. There is a significant difference between the mechanical properties of steel bars under dynamic and static loading. Steel has an obvious strain rate effect under dynamic load.

To describe the strain rate effect of steel bars under dynamic loading, scholars have proposed different constitutive models of steel bars considering the strain rate effect. Among them, the Cowper-Symonds^28^ model and the Johnson–Cook^29^ model are widely recognized by scholars. Once the strain rate is lower than a certain critical value, the growth of compressive strength decreases, and when the strain rate exceeds this critical value, the growth rate of compressive strength increases rapidly. Atchley and Furr^30^ conducted dynamic and static compression tests on concrete cylinders and found that the ultimate strength does not increase when the strain rate reaches a certain level.

Wu et al.^31^ and Yan and Lin^32^ found that the concrete tensile crack surface develops more straight under rapid variable loading, forcing the crack to pass through areas with greater resistance, such as aggregates. Therefore, a higher stress level is required to cause the specimen to fail. The same controversy still exists regarding the strain rate effect of concrete dynamic tensile strength. Cotsovos and Pavlovic^33^ believe that the increase in tensile strength at high strain rates is related to the structure's inertial effect rather than the material's actual behavior. Lu and Li^34^ proved through numerical simulations that the model did not increase strength due to the increase in strain rate. Therefore, the increase in concrete tensile strength observed in the dynamic test is the actual behavior of the material.

Liu et al. examined reinforced concrete members' dynamic response and failure mode. FRP reinforcement may modify the failure mode of members in both experimental and finite element analysis. After being wrapped in FRP layers, the shear failure of RC members becomes a bending failure^4^. Abas et al. studied the response of four RC square members to a lateral force. A finite element model was suggested to predict RC member impact responses. The results could improve RC members' withstand damage^3^.

Cai et al.^35^ used ABAQUS to simulate the dynamic response of 7 reinforced concrete members with a section size of 150 mm × 150 mm under low-speed horizontal impact loads. The authors studied the effect of impact mass and velocity on the failure mode of members. It is found that the inertia effect has a significant impact on the impact resistance of the member. Shen et al.^36^ utilized finite element modeling to analyze and reproduce the Bridge barge lateral impact accident and examine its causes. The FE findings suggested that the bridge collapsed due to flexural failure of the longitudinal pile foundation, which coincided with the field survey. Lateral impacts are worse than head-on and oblique crashes.

Furthermore, using the tested specimens and comparable specimens available in the literature^3,4^, the RC members' finite element (FE) models were developed and verified. The effects of the longitudinal reinforcement and stirrups ratio, impact velocity, concrete strength, and slenderness ratio on the dynamic response parameters of the RC members under unequal lateral impact loads were investigated further using the verified models. The study's findings will contribute to developing design codes for RC members subjected to unequal impact loads.

This research explores the numerical study of existing RC circular members' dynamic response and failure mechanism under unequal impact train collision to the experimental results reported in the author’s work^37^. For more clarity, Table 1 describes the specimens' concise properties. The deformations are considered during the simulation as observed in practical situations.

**Table 1:** List of concise specimen properties.

No	Longitudinal reinforcement ratio	Volume stirrup ratio	Impact high (m)	Notes
YH1	1.67% (6∅6)	1.26% (∅4@50)	1.0	Cross-section is 114 mm, and impact velocity is $v = \sqrt {2gh} , {\text{g}} = 9.81{\text{ m}}/{\text{s}}^{2}$, impact energy *E* = mgh; *m* = 270 kg
YH2	1.67% (6∅6)	1.26% (∅4@50)	2.0	
YH3	4.61% (6∅10)	1.26% (∅4@50)	2.0	
YH4	1.67% (6∅6)	0.63%(∅4@100)	2.0	

## Crack development

**Figure 1:**
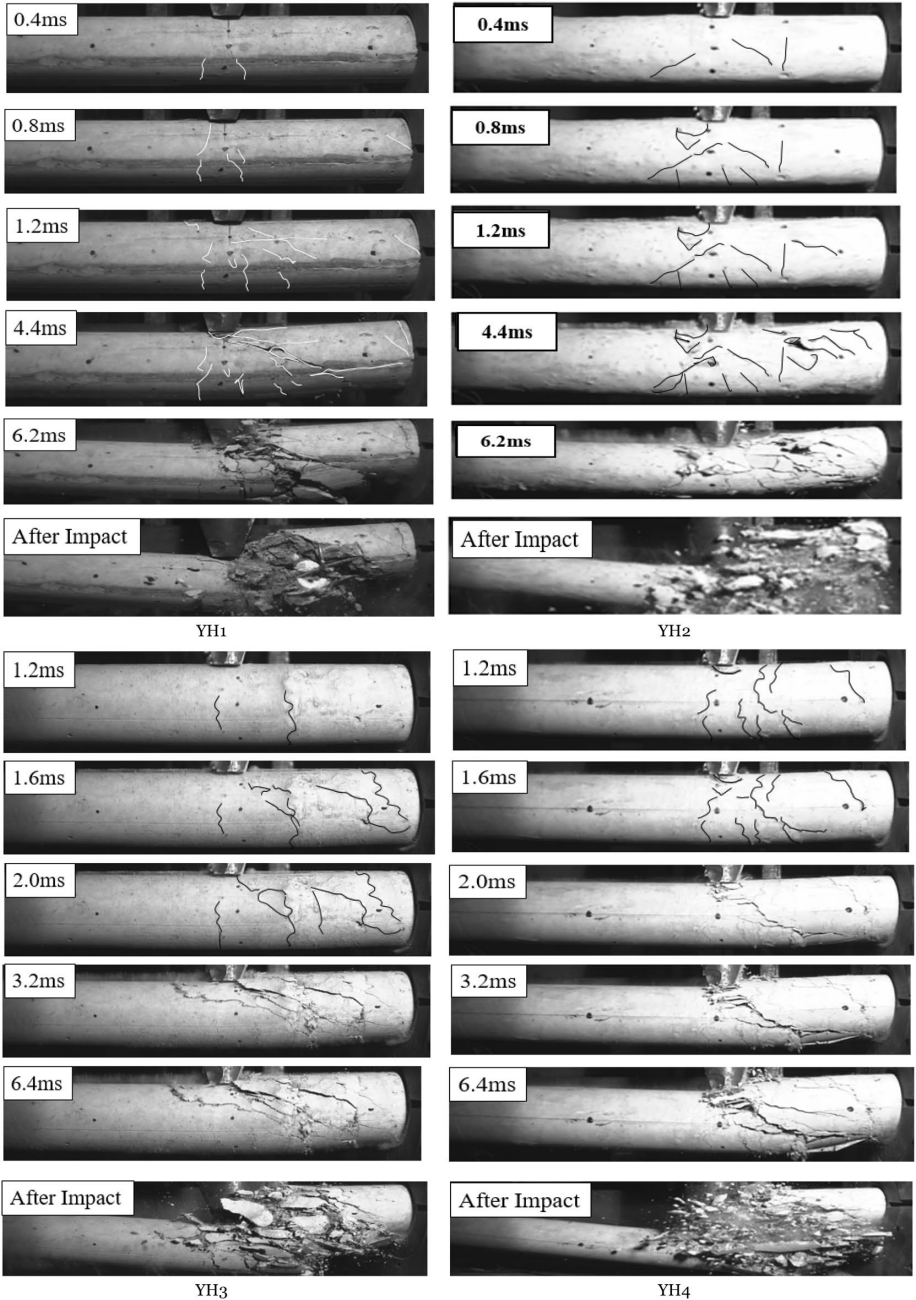
YH1 crack development process.

Starting with the impact body and test specimens and finishing with the specimens' damage, this part delves into each specimen's crack development process in-depth, using the different explanations of crack modes from the experimental paper^37^. The figures below depict the crack's development shape captured by a high-speed camera for each specimen and describe where the crack develops. Only the specimen's right side could be imaged due to the imaging distance limitation (i.e., short-span region). The early impact crack was difficult to observe, marked with arrows. The arrow pointed in the same direction as the crack. The high-speed camera documented each member's destruction in Fig. 1. As shown in the figure, all specimens fail under shear, with the top end of the failure surface near the impact point and the lower end depending on the specimen's characteristics.

YH1's failure surface is furthest from the near support's bottom. The specimen is severely damaged, but only between the impact point and the damaged surface. The failure surface of YH2 disappears entirely at the support's bottom, and the crack continues into the support. So, after the collision, the concrete is severely damaged and crushed, and the support's fixed part is pushed out for a distance. YH3's lower failure surface lies inside the shear span, closer to the support than YH1. The concrete from the right side to the failure surface collapsed, but the steel bars held firm. YH4 crack failure surface expanded, and the specimen's bottom concrete was split longitudinally. The concrete between the impact point and the support was shattered on the right side of the impact point. The steel bar pushes the support out a distance, crushing the concrete between the impact point and the support and seriously cracking the pushed-out concrete in the support. The failure surfaces of YH2 and YH4 disappear entirely at the support's bottom, and the crack continues into the support. Zhao et al.^38^ postulated that shear cracks might occur in three ways in slender beams with a significant shear forces ratio.

The impact height and reinforcement ratio may decrease specimen damage. Increasing the stirrup ratio has little influence on the initial impact on the specimen, but it may reduce the degree of concrete crushing. The experiment shows that the angle of the shear crack is related to the specimen's stiffness and impact velocity.

## Numerical simulation software

Take advantage of ABAQUS to simulate the outcomes of the experiment. It is mainly concerned with nonlinear dynamic analysis and provides functions for static analysis as a side effect. These fields mostly focus on structural analysis, which is well-known for its great accuracy in simulation and calculation and its ability to predict possible results.

### Material model

The finite element analysis program ABAQUS (revision 2020) was used to model the nonlinear behavior of unequal lateral impact load members. The primary objectives of the FEM are to introduce an alternative tool that may be used in analysis or design and to verify the experimental works. The finite element simulation consisted of three stages. The first stage defines the module, assembly, and mesh geometry. The second stage will consider all the materials modeling definition properties, interaction contact, and boundary conditions. Afterward, select the output finding fields according to the research parameters in the third stage.

**Figure 2:**
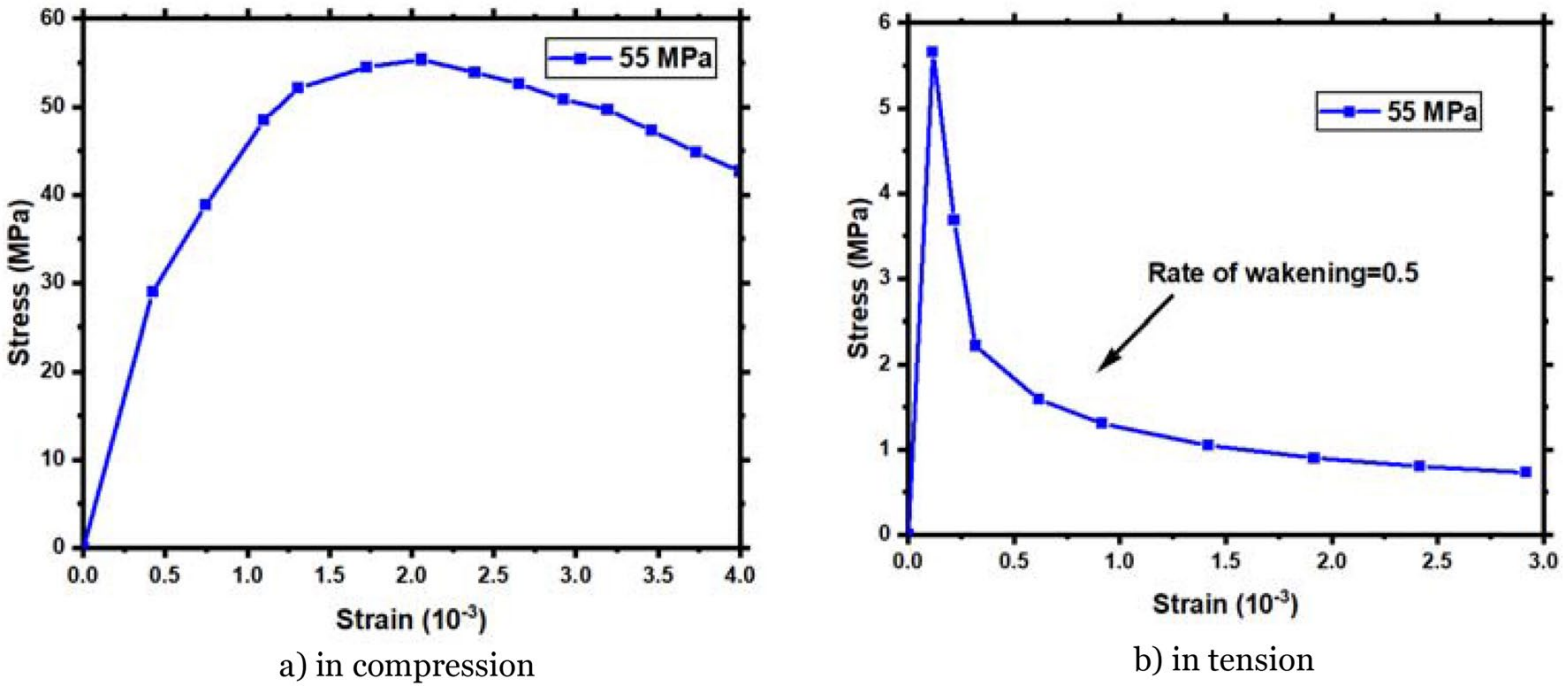
Stress–strain behavior of the concrete.

To simulate the behavior of reinforced concrete in this paper under dynamic loading conditions relies on the concrete damage plasticity (CDP) model, which has a ground-breaking record in the recently completed studies related to the development of original (CDP) models to determine the damage of reinforced concrete in the ABAQUS simulation software. The effect of mesh size on the stress–strain curves on the compressive and tensile behavior during the softening phase is considered (in the case stress reaches peak strength) for finite element models. The stress–strain curves in the CDP model have many advantages compared with the recently published results of previous studies. Simultaneously, an exponential function is used to replace the values of the tensile damage (*d*_*t*_) and compression (*d*_*c*_) variables mentioned in previous studies^39–43^. The dynamic cases have the specificity of using the Dynamic Increase Factor (DIF) according to what was mentioned in fib MODEL CODE 2010 (MC2010)^44^ to determine the strain rate effect on the compressive strength of reinforced concrete. To establish the reliability and effectiveness of the proposed model, numerical simulation of dynamic compressive tests is implemented. The study's CDP model agrees with experimental data for dynamic loading circumstances with significant reliability^43^. Concrete damaged plasticity (CDP) is a prominent ABAQUS software material model for plain and reinforced concrete. Lubliner et al.^45^, Lee and Fenves^46^, and others characterized it. This model requires values of some material constants. Correct material constant values are an open scientific question^47^. The use of software while performing impact and explosion research is essential. It allows researchers to better illustrate the damage and destruction process of concrete under high-speed impact while also considering the strain rate effect of the concrete^48^. Figure 2a shows the compression stress–strain curve using Saenz's constitutive model^49^. The hardening and softening branches are assumed to follow a parabolic trend in this constitutive model by Saenz. Tension behavior is studied using Hsu and Mo^50^ exponential tension stiffening curve, as reported in Abas et al.^3^. The RC member's weakening factor (n) is 0.5, which is based on the stress–strain behavior of the concrete, as illustrated in Fig. 2b.

The element types in ABAQUS software are mentioned in Table 2, and the input parameters in Table 3. This paper is a part of a big project with different details of shapes and specimen dimensions. Nevertheless, the concrete and steel constitutive model definition, strain rate effects, and modeling methods are similar to the author's previous work^3,24^.

**Table 2:** Element type in ABAQUS software.

Material	Element type
Concrete	C3D8R
Reinforcement bars and stirrups	T3D2
Hammer	R3D4

**Table 3:** The input parameters in ABAQUS software.

General	Density (g/mm^3^)	2.3
Elastic properties	E (N/mm^2^)	36,896
	Poisson's ratio	0.18
Plasticity parameters	*ϵ*	0.1
	*ψ*	31
	*σ*_*b*0_/*σ*_*c*0_	1.16
	*K*_*c*_	0.667
	Viscosity	0
**Concrete behavior**		
Compressive behavior	Yield stress *σ*_*c*_	The compression data are shown in the author's previous work^24^
	Inelastic strain $\varepsilon_{c}^{in,h}$	
	Damage parameters *d*_*c*_	
Tensile behavior	Yield stress *σ*_*t*_	The tension data are shown in the author's previous work^24^
	Inelastic strain $\varepsilon_{t}^{ck,h}$	
	Damage parameters *d*_*t*_	

### Element type, meshing, and boundary conditions

**Figure 3:**
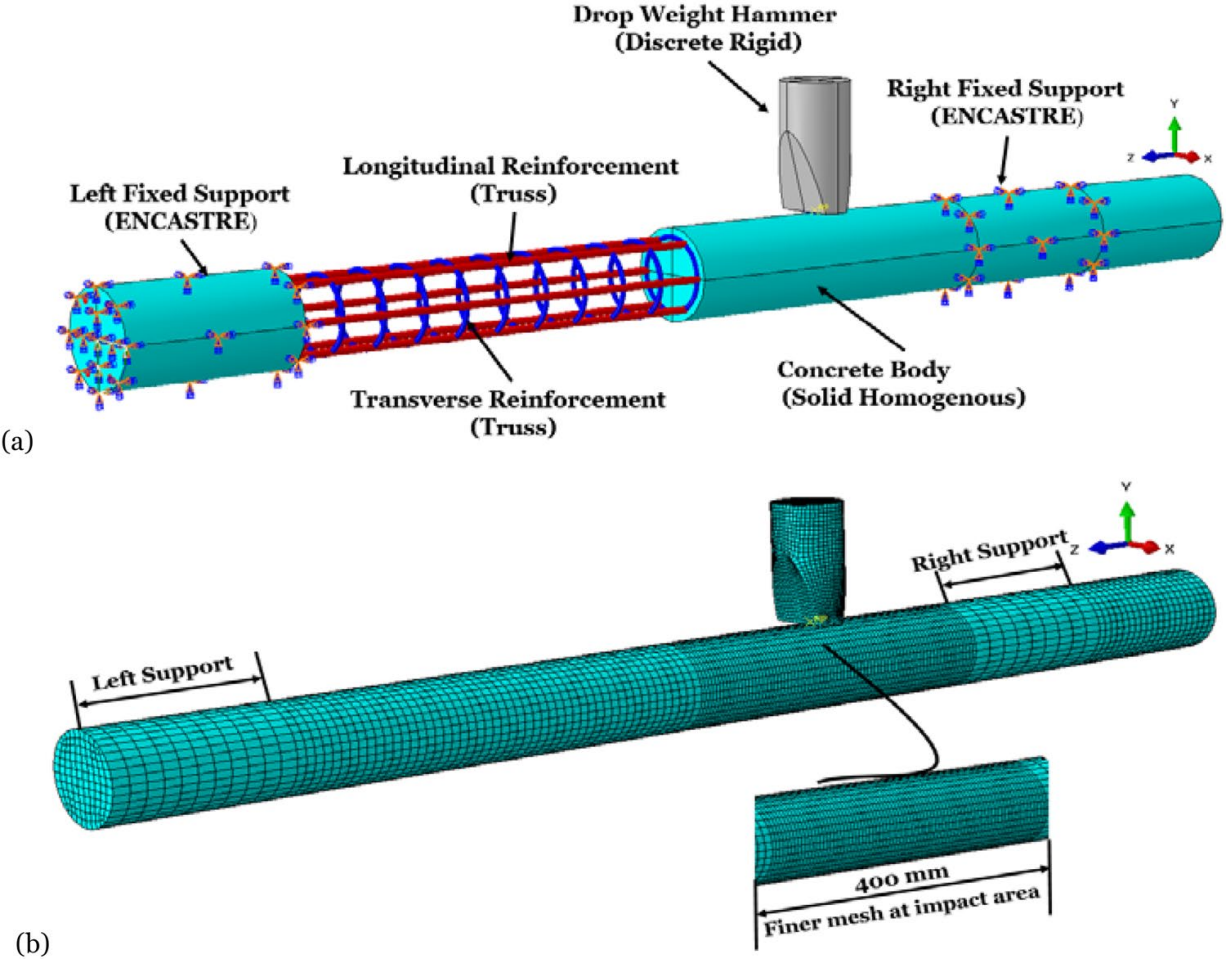
(**a**) FEM modeling assembly; (**b**) FEM meshing.

Figure 3a shows the models of concrete, steel bars, stirrups, impact bodies, and boundary supports developed in ABAQUS for structural analysis. Hexahedral meshes are used to split all of the models. The density of the finite element grid significantly impacts the accuracy of the calculations. However, although it is commonly acknowledged that a denser grid produces a more accurate numerical simulation, the dense grid is very time-consuming to construct.

**Figure 4:**
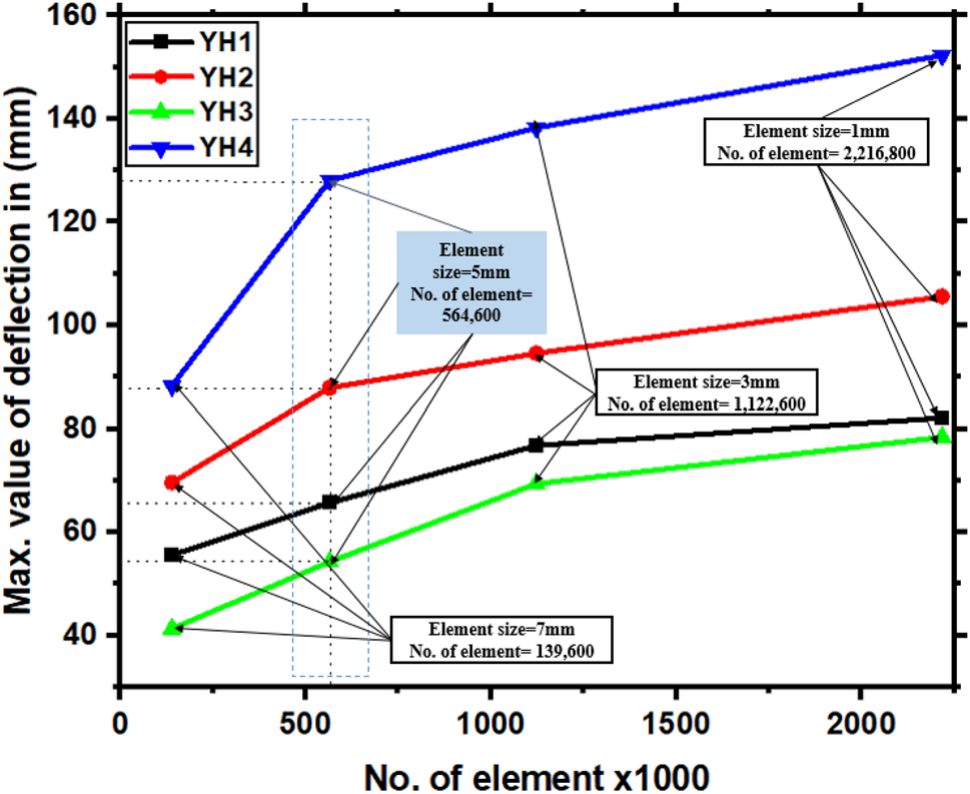
Mesh convergence analysis.

Therefore, convergence is essential to the accuracy of solutions obtained by numerical models. When adopting explicit FEA techniques, coarse meshes might provide inaccurate analyses^3^. Due to the sensitivity of the mesh density, which significantly influences the outcomes of the numerical model, four different mesh densities are tested, and there is no convergence between the findings. Thus, after a convergence analysis test, the concrete's element edge length was set to 5 mm in both the length and radial directions. The results of the mesh density study as illustrated in Fig. 4. The data was collected using various devices, and the FE analysis was performed on a computer equipped with Intel i9 processors, as previously indicated (CPUs). According to the software, the anticipated wall-clock time necessary to replicate a 0.06 s impact scenario in ABAQUS with an interval of 6000 was around 6 h. Therefore, the meshes in the finite element model presented in this work are locally refined around the area of impact, which is located in the longitudinal direction of the member, as shown in Fig. 3b. To guarantee that the three materials of concrete, longitudinal reinforcement, and stirrups are always in a single node state throughout the impact process. The grid density of the longitudinal reinforcement, concrete, and stirrups must be the same along the length direction of the member. On the other hand, the remainder of the mesh is a lot more sparsely distributed. The drop hammer deforms very little during the impact, which is close to a rigid body, so it is considered a rigid cube in the finite element simulation^1,3,36,51^, with a cross-sectional size of 80 mm × 30 mm, consistent with the cross-sectional size of the impact body in the test. As a result of this treatment, the analysis time required for the drop hammer contact to RC collision simulations can be reduced by 43%. The fixed-end support is also close to a rigid body in the test, so it is considered a rigid circular sleeve in the finite element model with a thickness of 30 mm. For both sides of the member, ENCASTRE (which mean fully built-in (U1 = U2 = U3 = UR1 = UR2 = UR3 = 0)^52^) set the boundary conditions as fixed support. All element nodes on the support sleeve should have translational and rotational degrees of freedom constrained in the X, Y, and Z directions. A free fall from a 2 m high rail was used in the test, and the drop weight came into contact with the member at that point. It is possible to reduce computation time and file size by having the falling weight fall 0.1 mm away from the impact location and then simulating how the weight impacts by giving an initial velocity for the falling weight in the software.

### Contact definition

Numerical simulation encounters another significant hurdle in the modeling of contact definition surfaces. The contact interaction algorithms in ABAQUS/Explicit are divided into two categories^3^. The definition (SURFACE_TO_SURFACE_CONTACT) is adopted between the member and the falling weight. This option employs advanced detection algorithms to maintain contact. The PURE MASTER SLAVE HARD kinematic algorithm simulates the contact behavior between rigid and deformable bodies. Compared to quasi-static stress, the final dynamic bond at failure is 70%–100% higher^53^. Steel deformation is a little below the impact point^54^. The time required to cause a considerable bond slip down the steel bar is inadequate. During the impact process, the slip between the steel bar and the concrete is very small. It has little effect on the impact dynamics of the members, so it is considered via a technique whose response is used to constrain the translational degrees of freedom of the embedded nodes; this constraint technique is called EMBEDDED_ELEMENTS. The overlay approach introduces position constraints insofar as nodes of the concrete part and the reinforcement part coincide^55^.

## FEM model validation

The ABAQUS model parameters were validated numerically on four specimens. The structure's failure mode, impact force, and deflection-time history curve are validated.

### Member failure modes

**Figure 5:**
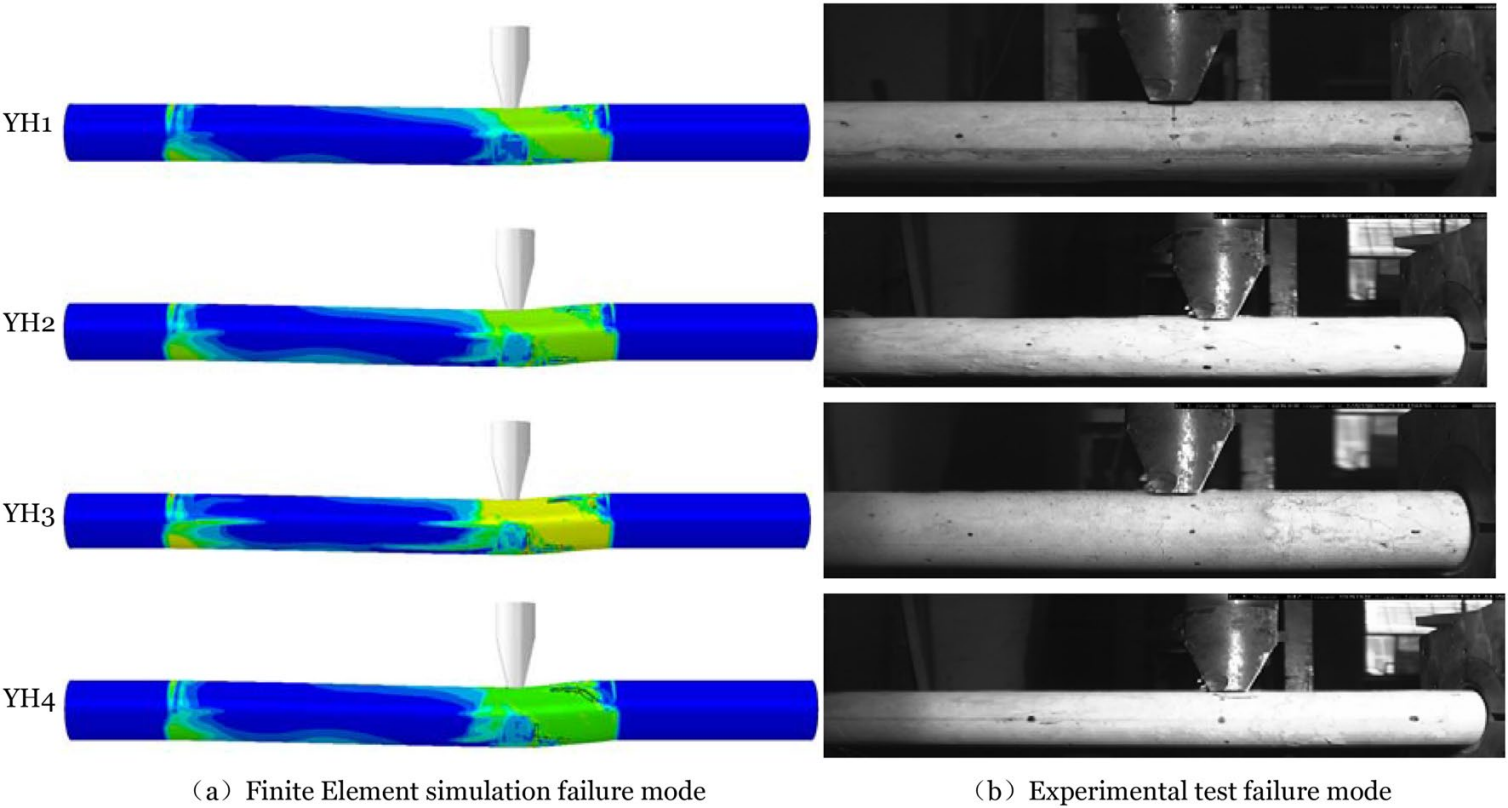
Compares the failure modes chart of the 0.8 ms test process.

**Figure 6:**
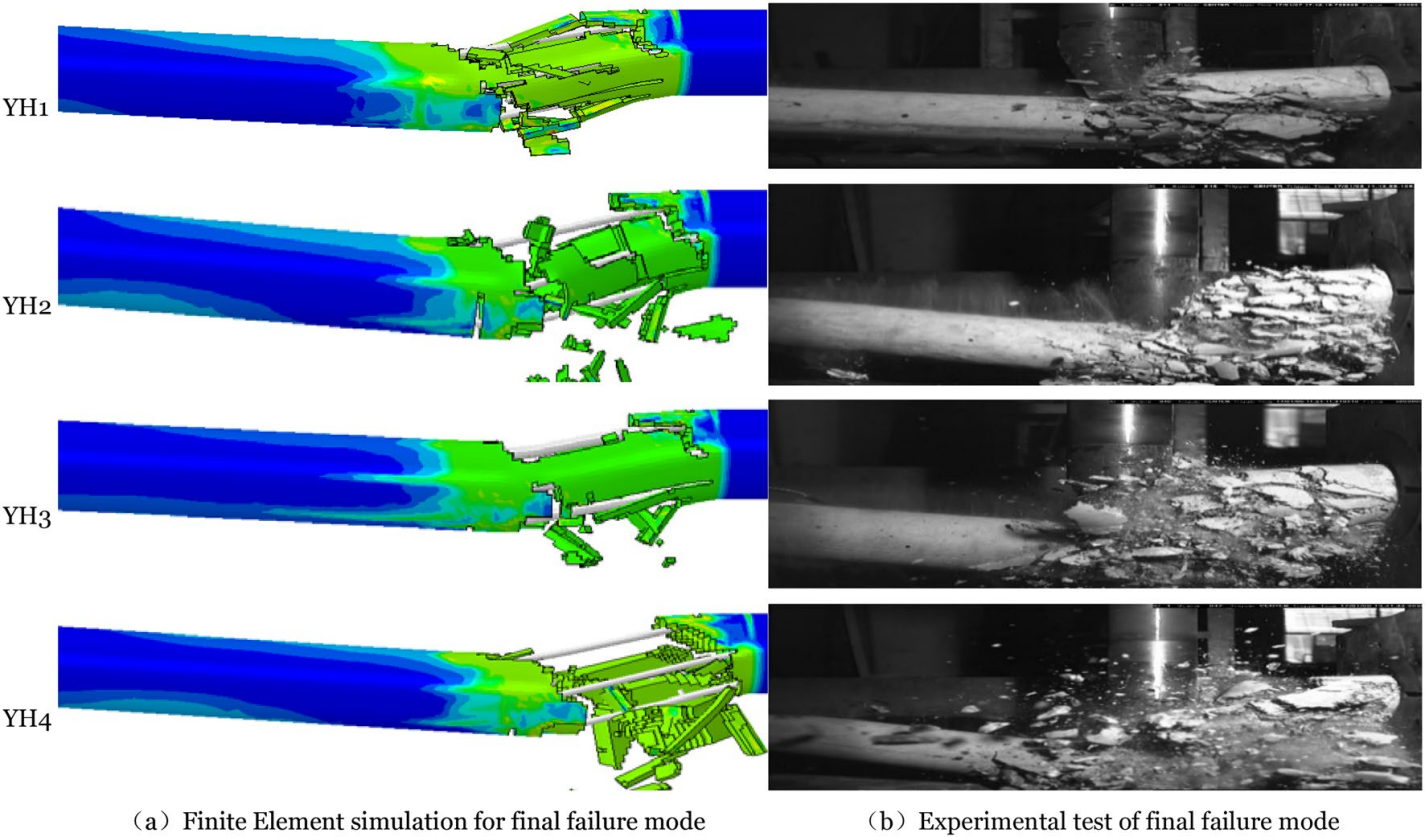
Compared the final failure modes.

Figure 5 compares the member's strain diagram at 0.8 ms to the experimental behavior at 0.8 ms produced using the finite element technique. Figure 6 compares the final failure mode of the member. At 0.8 ms, the greatest member strain region in the figure matches the test's most severe concrete damage development area, represented by bending and shear failure at the member's impact point and bending failure at the right end support and impact point. The final failure mode demonstrates that shear failure dominates all members promptly after impact. Severe damage occurs between the impact point and the failure surface. It demonstrates that the finite element model is more accurate.

### Member deflection and impact force

**Figure 7:**
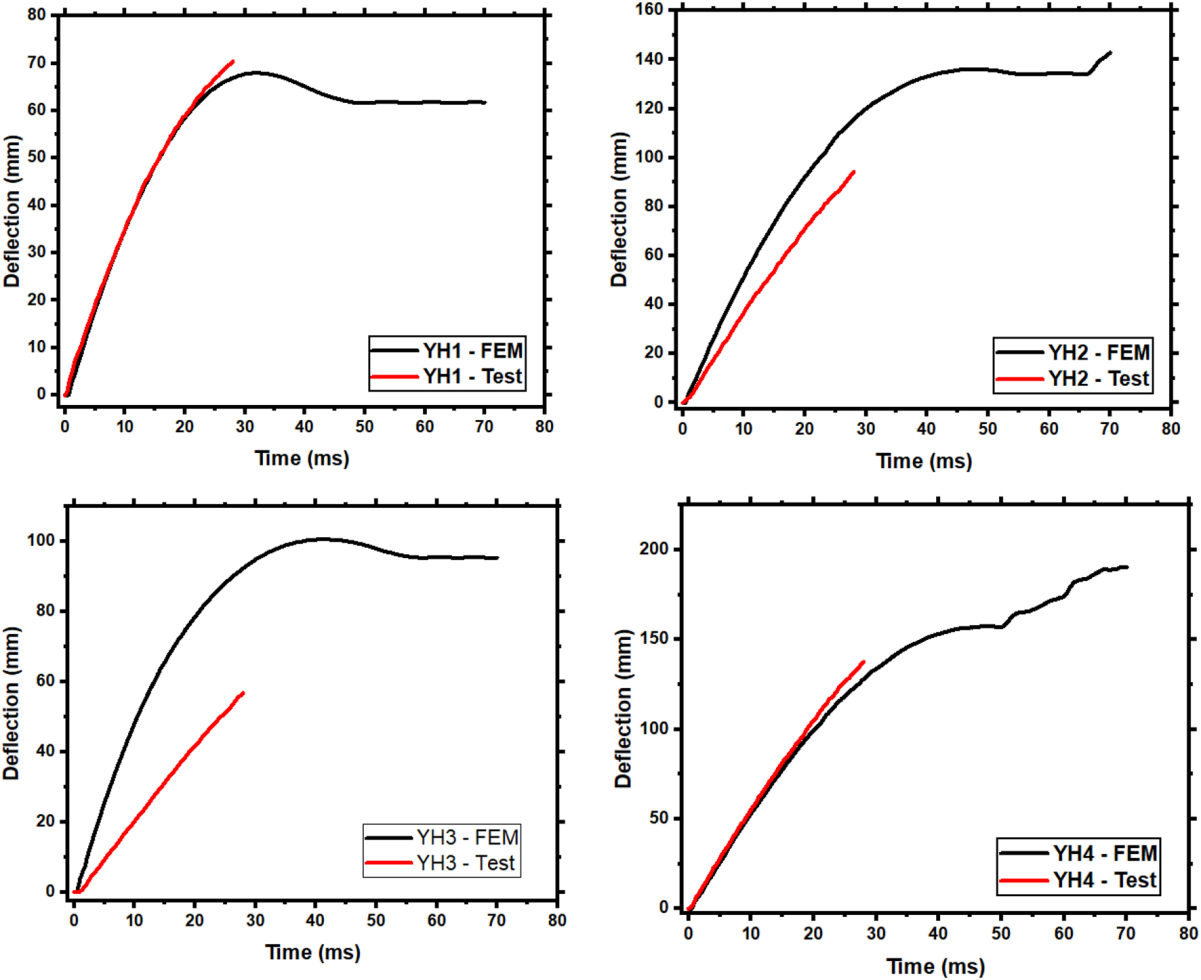
Comparison of deflection time history curves.

**Figure 8:**
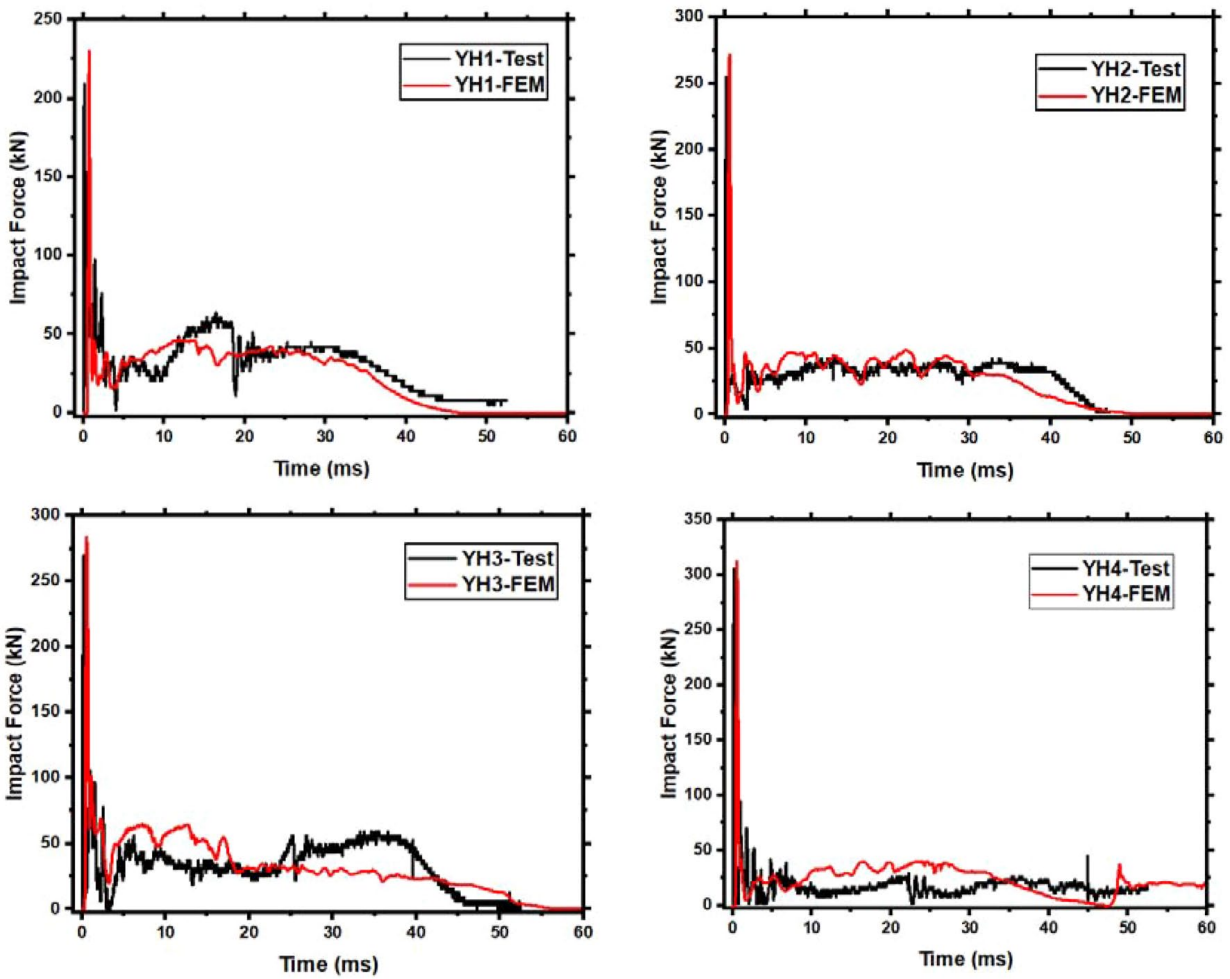
Comparison of impact force–time history curves.

Figure 7 compares the experimental and simulated values of the member's deflection time history curve. The contrast between the test value and the simulated value of the member impact force–time history curve is shown in Fig. 8. As demonstrated in Fig. 7, the collecting time for all members in the test is substantially faster than the simulation. Since the deflection collection point is exactly below the concrete impact point. The concrete below the impact point and on the right side of the member has peeled or fractured, resulting in an incomplete deflection time history curve during the test. When the experimental deflection data cannot be gathered, the experimental deflection value is compared to the simulated deflection value to compute the relative inaccuracy. Table 4 shows the computation result. The findings reveal that each member's deflection change rate is the same before the test data collection fails.

**Table 4:** Deflection time history comparison between FEM and test values.

No	Member deflection time history			
	Test final deflection	FEM value	Final displacement of FEM value	Error (%)
YH1	70.39	65.69	68.01	6.68
YH2	94.30	87.93	136.15	6.76
YH3	56.77	54.31	100.62	4.33
YH4	137.55	128.10	174.15	6.87

$$Error\,\left( \% \right) = \frac{Test\,Value - FEM \,Value }{{Test\,Value}}$$.

Each member's impact force–time history curve shows the same changing pattern in Fig. 8. Comparing the test peak and plateau values to the simulated values yields a relative error. Results are displayed in Table 5. The findings reveal that the inaccuracy is less than 15%, acceptable. Depending on the aggregate thickness around the impact site, the local stiffness may fluctuate, causing the peak impact force to differ slightly. However, the concrete and steel bar components will be invalid and eliminated using finite elements. This configuration is not accurate, producing the impact force plateau errors.

**Table 5:** Impact force comparison between FEM and test values.

No	*F*_*peak*_ (kN)			*F*_*plateau*_ (kN)		
	Test value	FEM value	Error (%)	Test value	FEM value	Error (%)
YH1	209.30	229.89	9.83	39.55	37.29	5.71
YH2	255.30	268.12	5.02	34.18	39.10	14.39
YH3	269.77	283.44	5.06	40.16	39.88	0.70
YH4	305.43	312.31	2.25	16.20	16.39	1.17

Reinforced concrete members subject to lateral impacts are modeled using finite elements in three dimensions in this section. Concrete, steel bars, and contact between parts are all considered in the model. Simulating the failure of each tested part, the concrete three-dimensional solid element is controlled by maximum main strain, whereas a steel bar is controlled by effective plastic strain. The model's failure mechanism, impact force, and deflection time history curves were confirmed. The findings demonstrate that the finite element model developed in this study adequately predicts reinforced concrete members' mechanical characteristics and failure mechanisms under lateral unequal-span impact loads. This model may be used for further study.

## Parametric study effects on the dynamic response of RC members

The test findings describe reinforced concrete members' failure modes following an unequal span impact and force characteristics. The effect of numerous variables on the members' impact resistance is not well known because of the limited number of test members. Section FEM model validation demonstrates the validity of the finite element model and explains why it is appropriate. The variables in the test are evaluated together with the finite element model to understand better the dynamic response of reinforced concrete members under the lateral unequal high-speed impact and the aspects that were not included in the test. To analyze these parameters, one variable is altered. Some variables to consider are steel ratio, concrete strength, impact velocity, stirrup ratio, and slenderness ratio.

### Effect of steel ratio

Increasing the longitudinal reinforcement ratio of concrete members within a range may enhance stiffness, minimize fracture appearance, and reduce crack propagation. Increasing the longitudinal reinforcement ratio to (2.76) times the original may significantly decrease concrete crushing and damage. It is shown here how altering the longitudinal reinforcement diameter changes the reinforcement ratio of the members and how this affects their performing mechanism. According to the experimental test data^25^, there are two types of longitudinal reinforcement ratios, YH1, YH2, and YH4, with 6ø6 (reinforcement ratio of 1.67%). Another type is YH3 with 6ø10 (reinforcement ratio of 4.61%). Four longitudinal reinforcement ratios of 6ø8, 6ø12, and 6ø14 were also considered. The longitudinal reinforcement ratios are 2.95%, 6.65%, and 9.05%, respectively. The members with different longitudinal reinforcement ratios indicate as LR1 (6ø6), LR2 (6ø8), LR3 (6ø10), LR4 (6ø12), and LR5 (6ø14), according to the different diameters of steel bars.

**Figure 9:**
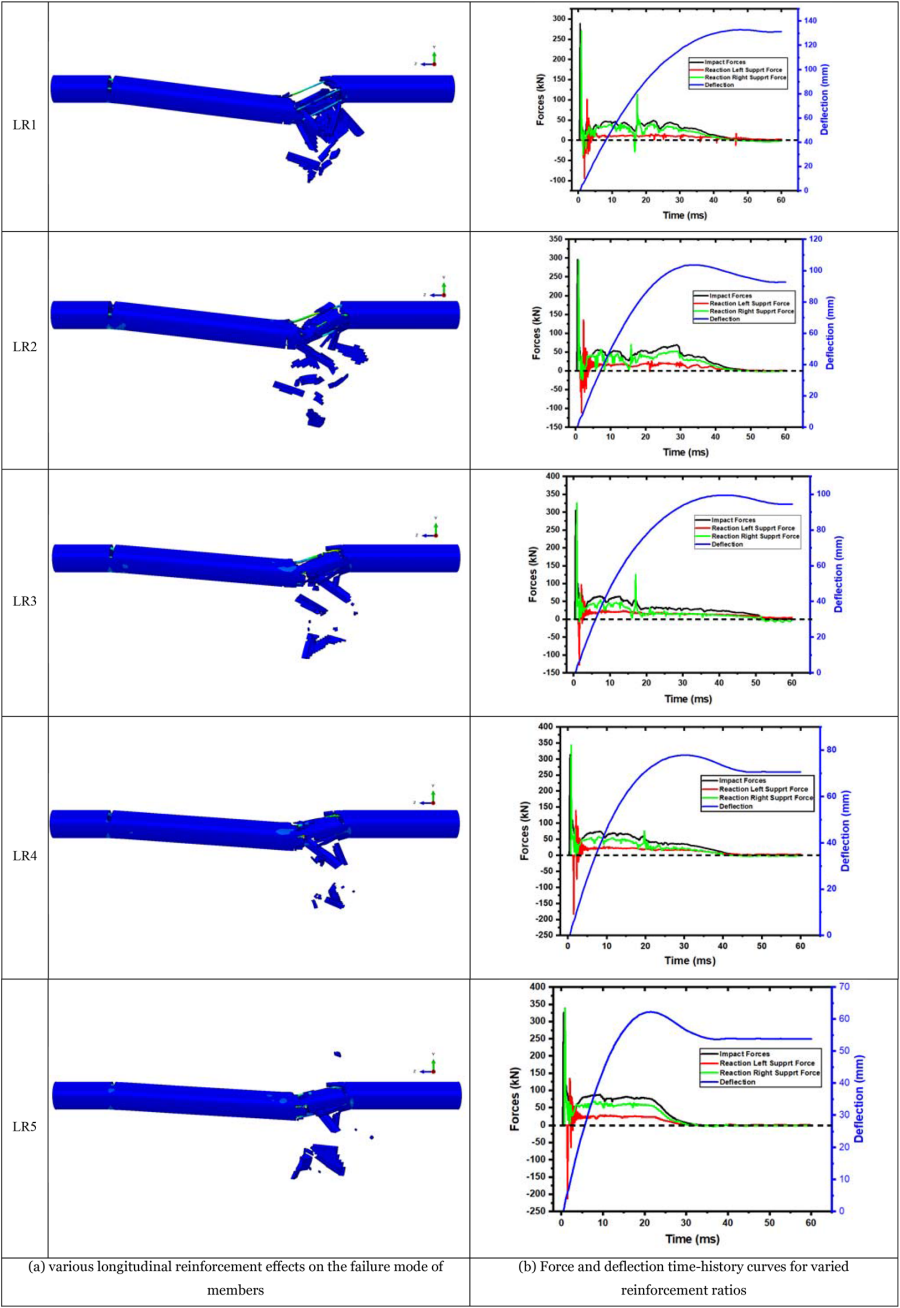
Force and deflection time-history curves with failure mode for varied reinforcement ratios.

Figure 9a depicts the failure mode of members with various longitudinal reinforcement ratios following impact loading. The figure shows that all members shear when subjected to unequal impact loads and that changing the longitudinal reinforcement ratio does not affect the members' failure mechanism. The longitudinal reinforcing ratio increases the member damage range and brings the damaged concrete section closer to the impact point. Horizontal tensile cracks and shear oblique fractures formed exclusively on the top steel bar deflection on the right side of the impact point when the longitudinal reinforcement ratio increased to 9.05%. This observation demonstrates that increasing the longitudinal reinforcement ratio may enhance total member stiffness. Increasing the longitudinal reinforcement ratio reduces the energy absorbed by the concrete by efficiently transferring impact energy to the supports at both ends.

Consequently, the concrete is less damaged, and the members are more stable. Figure 9b depicts the member's impact and reaction forces. There is a difference in reaction force between the left and right supports. The time to support is delayed, consistent with the unequal impact force position. The support reaction force on the left side develops linearly with the longitudinal reinforcement ratio. Increasing a member's longitudinal reinforcement ratio enhances the energy transfer rate as the right end supports the reaction force. The support reaction force increases with the longitudinal reinforcement ratio below 6.65% and decreases beyond 6.65%. Once the longitudinal reinforcement ratio exceeds a specific limit, the concrete is crushed. During the plateau phase, the right support reaction force fluctuates. After concrete breaks, steel bars redistribute stress to the failure mode surface. The member's deflection time history curve is shown in Fig. 9b. The timing of the greatest deflection occurrence is synchronized with the impact force decrease. The passage of time will be accelerated by increasing the longitudinal reinforcement ratio while decreasing the maximum deflection and final member deflection.

**Figure 10:**
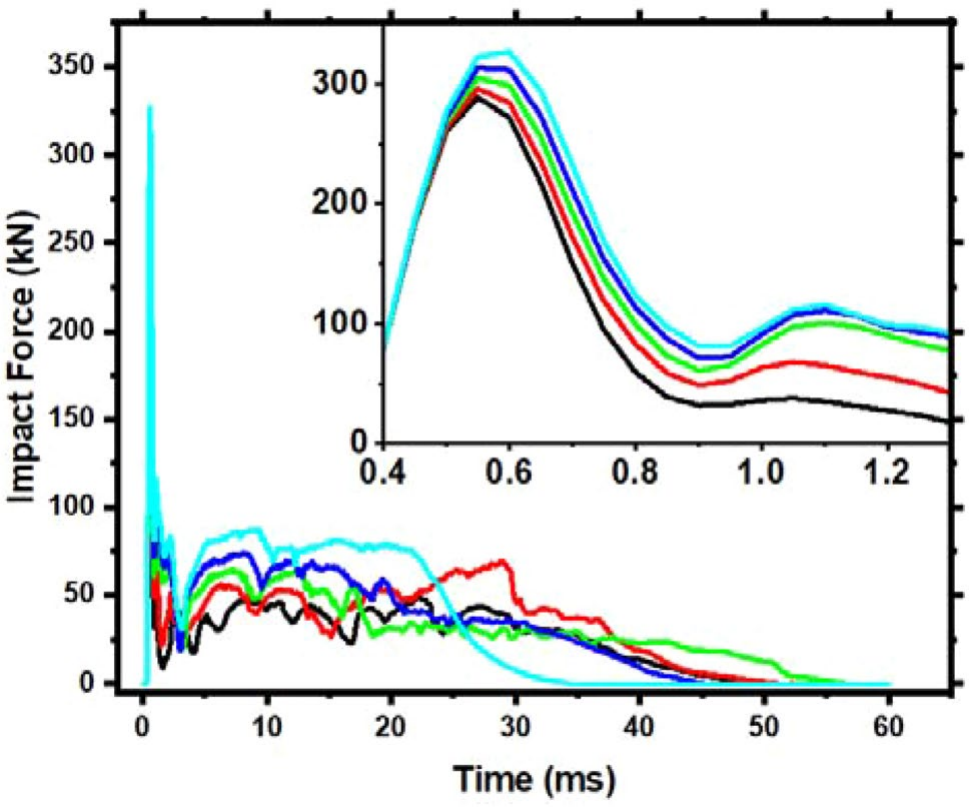
Impact force time-history curve for members with various longitudinal reinforcement ratios.

Details on each member's time history impact force curves are shown in Fig. 10. As can be observed, increasing the longitudinal reinforcement ratio significantly influences peak impact force, plateau value, and total member duration but not plateau duration. The peak impact force and plateau value increase linearly with longitudinal reinforcement diameter. This is due to the increased longitudinal reinforcement ratio. In addition, the member can swiftly transmit and absorb energy after being struck, improving total stiffness and inertial force.

**Figure 11:**
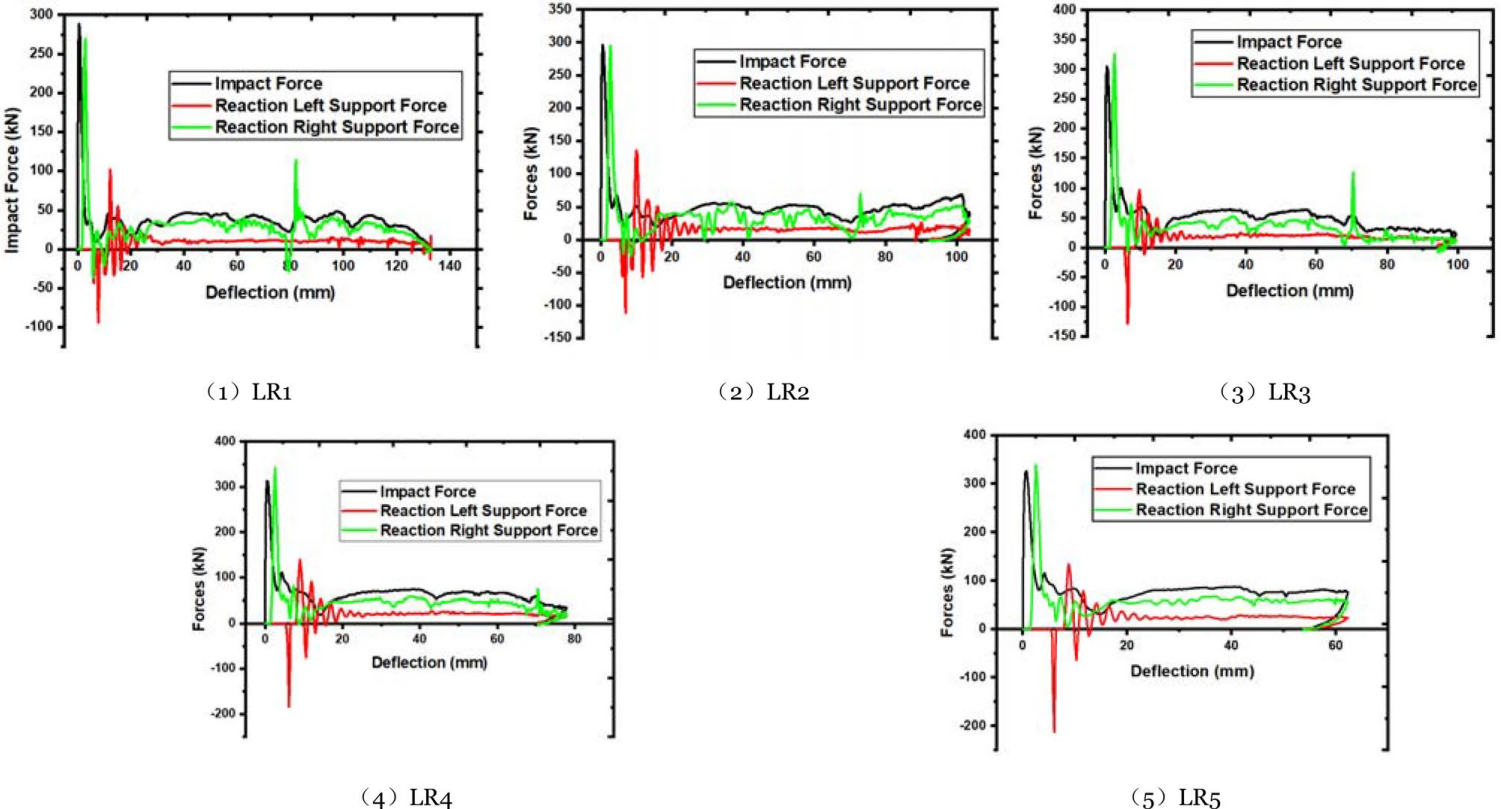
Force–deflection curve of members with different longitudinal reinforcement ratios.

The member's force–deflection curve is shown in Fig. 11, and the relevant statistical data for different longitudinal reinforcement ratios are presented in Table 6. Once the longitudinal reinforcement ratio is less than 6%, most of the energy absorbed by the member is transmitted to the right side support. The longitudinal reinforcement ratio increases the member's energy absorption capability. The member's energy absorption capability decreases when the longitudinal reinforcement ratio approaches 6%. Local concrete fracturing reduces the member's bearing capacity when the longitudinal reinforcement ratio increases and the steel is ineffective. The member's energy absorption is deducted from the support's energy consumption. The difference decreases when the reinforcement is less than 6% and increases when the reinforcement is larger than 6%. This occurrence eloquently demonstrates the preceding point of view.

**Table 6:** Relevant data statistics of members with different longitudinal reinforcement ratios.

No	Peak (kN)	Plateau (kN)	Left support (kN)	Delay (ms)	Right support (kN)	Delay (ms)	Deflection (ms)	Energy absorbing (J)	Left energy absorption (J)	Right energy absorption (J)
LR1	288.59	39.49	− 94.02	1.25	268.97	0.30	132.80	5043.10	1190.37	3758.58
LR2	295.95	39.10	− 111.21	1.15	294.81	0.30	103.60	5061.70	1486.22	3524.44
LR3	304.54	55.16	− 128.10	1.00	325.55	0.30	99.49	5114.49	1696.02	3390.55
LR4	313.24	63.47	− 183.86	0.95	343.18	0.30	77.83	5037.25	1451.58	3545.52
LR5	326.52	78.81	− 212.82	0.90	339.39	0.25	62.25	4893.79	1288.65	3528.14

The downward direction of the impact is positive, and the upward direction of the reaction force is positive.

A higher longitudinal reinforcement ratio increases the overall stiffness and hence the deformation resistance of a reinforced concrete member. However, once the longitudinal reinforcement ratio reaches a limit, the member's energy absorption capability is reduced owing to premature concrete collapse. The member's impact resistance decreases when the longitudinal reinforcement ratio exceeds 6.65%.

### Effect of concrete strength

**Figure 12:**
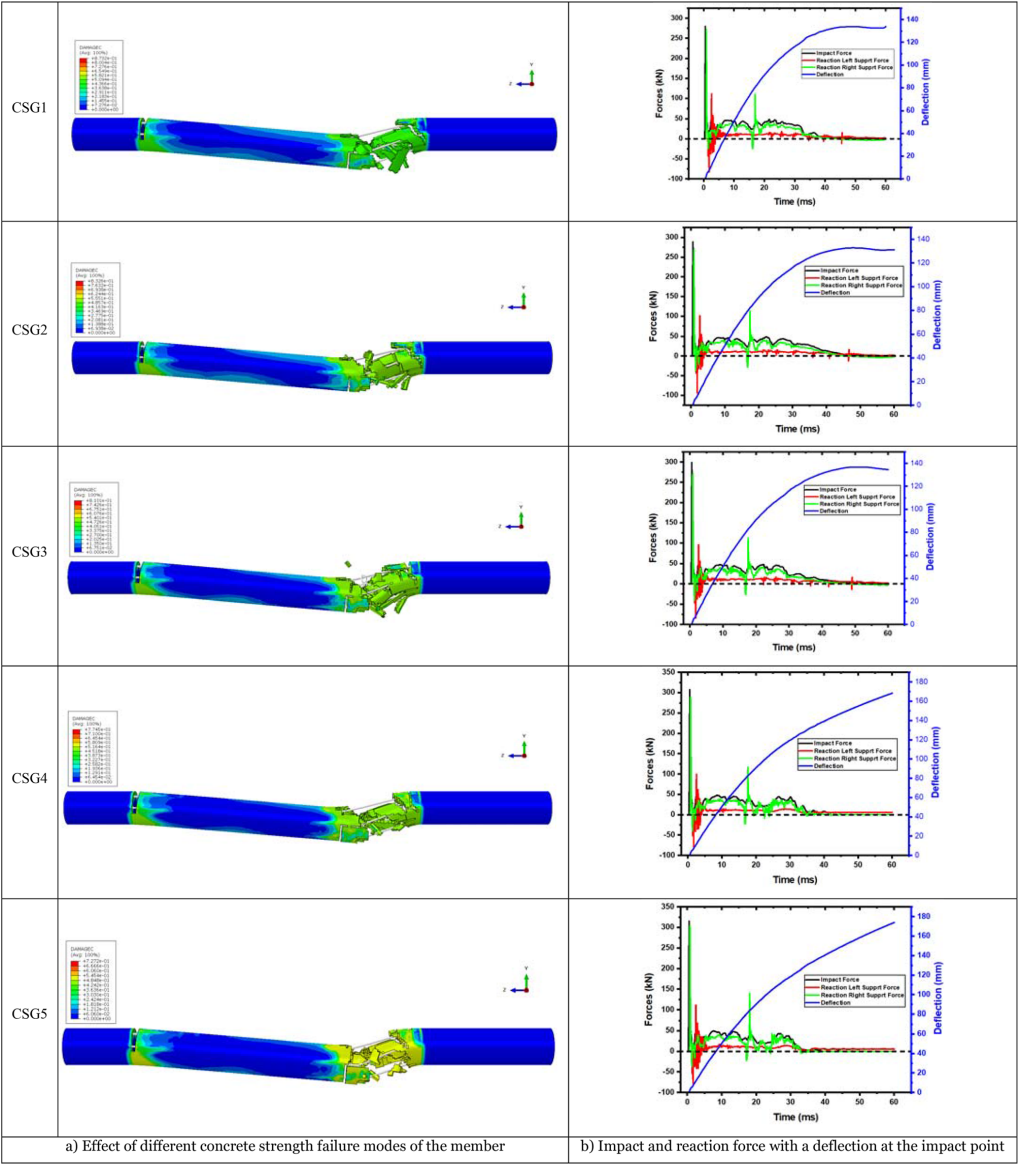
Failure mode and force–deflection time history curves of various concrete strength members.

The major role of concrete in reinforced concrete structures is to withstand compression, and the conventional values of compressive strength of concrete have changed dramatically. Thus, changes in concrete strength grade may affect the impact resistance of reinforced concrete elements. Concrete grade C55 was utilized in the experiment. Finite element simulations were used to add members with different concrete strength grades (C45, C65, C75, and C85) to examine how concrete strength affects the member's impact resistance. CSG1 (C45), CSG2 (C55), CSG3 (C65), CSG4 (C75), and CSG5 (C85) are indicated concrete strength grades as the five members with various concrete strength grades. Under varying concrete strength values, a member’s ultimate failure mode is shown in Fig. 12a. The illustration shows that shear failure occurs on the right side of the impact point, regardless of concrete strength.

For C65 and below, the concrete is shattered and split on the right side of the impact point owing to shear fractures, and the members split into two sections, joined by exposed longitudinal steel bars in the center. Tensile force is concentrated around the impact point. However, when the concrete strength exceeds C65, the longitudinal steel bars break on the right side of the impact point, indicating that the concrete becomes more brittle. A member bearing capacity is reduced when it is damaged. Excessive impact energy will rupture the longitudinal steel bar. Damaged concrete members during unequal impact increase as concrete strength increase above C65. Figure 12b shows the time history curve of each member's impact force and supporting response force with deflection. The arrival time of the first wave peak of the member's left and right end reaction forces is shortened as concrete strength increases, although the impact is slight. For the left end support, the reaction force fluctuates in the range of CSG5, which increases with concrete strength. It decreases with concrete when it exceeds CSG5. In the right end support, the response force has an upward tendency. For instance, higher concrete strength levels have higher compressive and tensile strengths and better resistance to damage within a range. It becomes brittle, and the fixed support's influence generates a compression, making the nearby concrete easier to fracture; each member's deflection time history curves as shown in Fig. 12b. The maximum deflection at the bottom of the impact point shows that changes in concrete strength do not influence the displacement trend at the member's impact point. Because concrete is excessively brittle, it will wholly fracture on the right side of the impact point.

**Figure 13:**
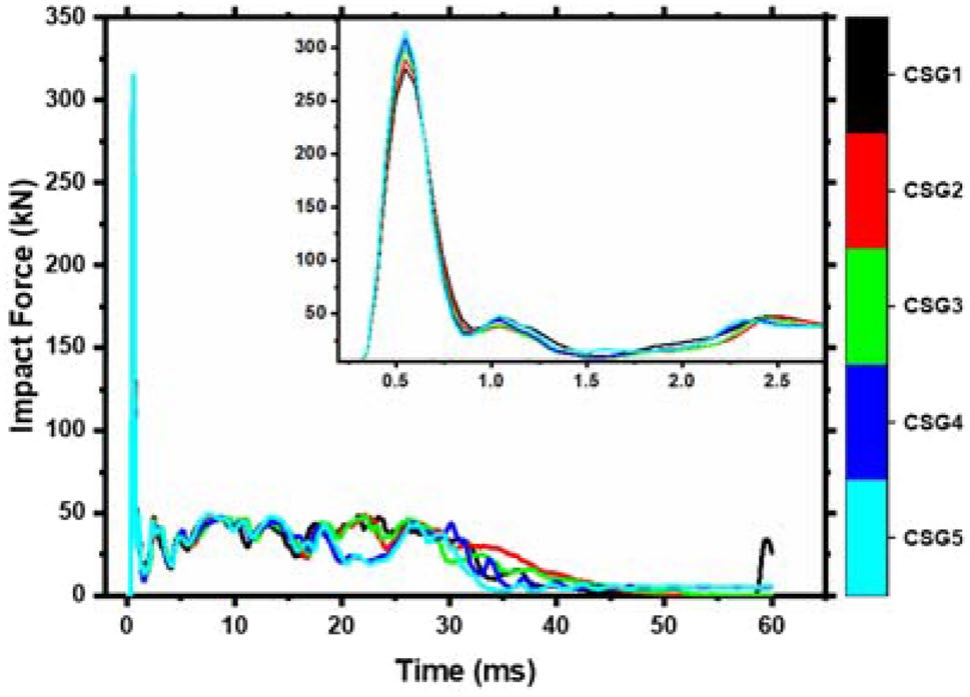
Time history of impact force of various concrete strength members.

Each member's impact force is shown in Fig. 13 to compare the time history curves when the concrete strength is variable. CSG1 and CSG2 impact time history curves nearly match, impact time history curves of CSG4 and CSG5 are practically common, and effect durations for all members are the same, as shown in Fig. 13. Plateau stage members CSG4 and CSG5 are fluctuating. Due to early concrete fracture and the abrupt decrease in the members' load-bearing capacity has occurred. At this point, the stress is distributed to steel bars, and their impact forces increase. The maximum impact force rises; however, the increase is modest.

**Figure 14:**
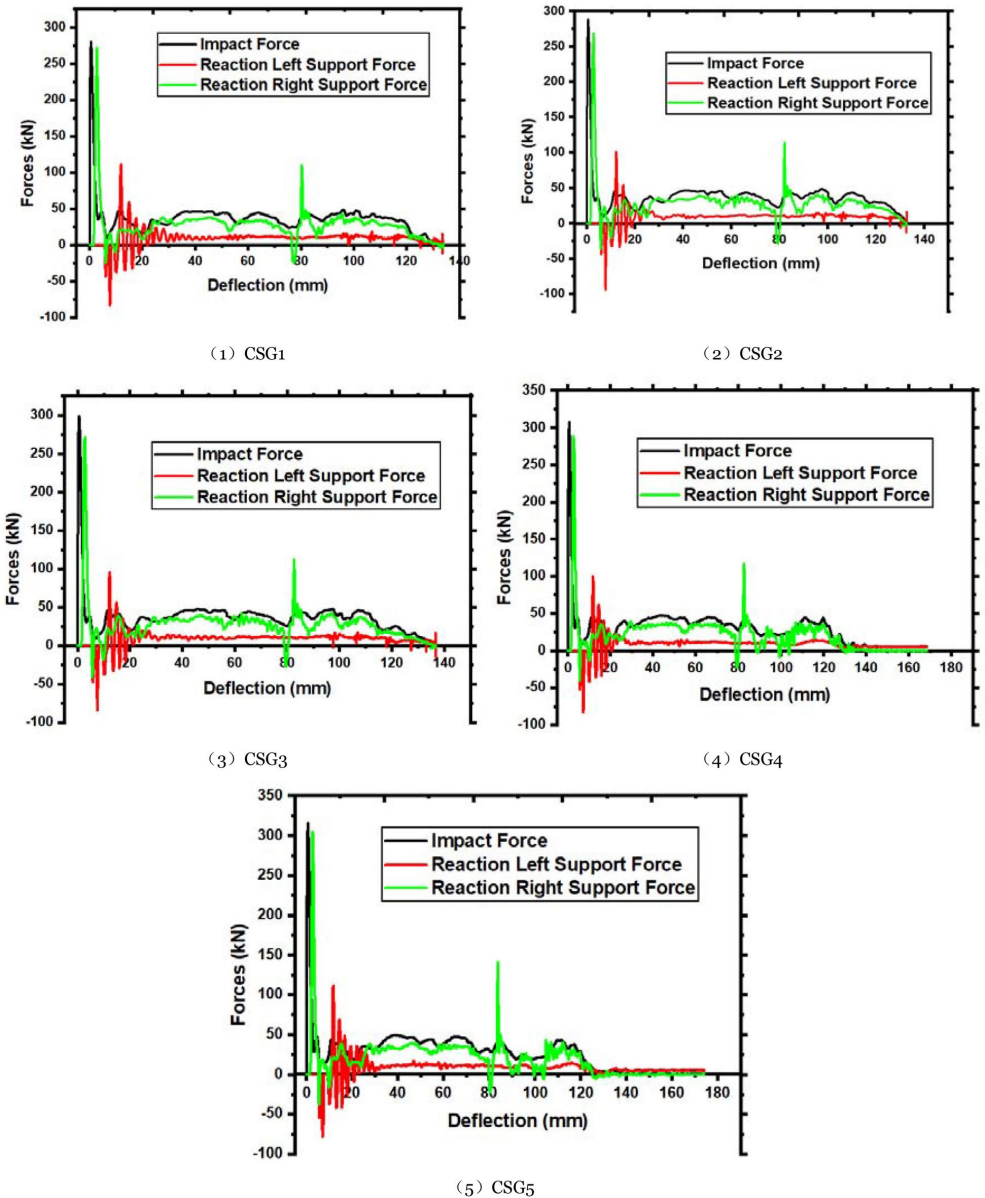
Force–deflection curves of different concrete strength members.

The force–deflection curve for various concrete strengths is shown in Fig. 14. It is integrating the curve yields the energy absorbed by the members. Table 7 details different concrete strength members' numerical data. Less than C65 concrete shows an increase in energy absorption by members with increasing concrete strength grade, as seen in Table 7. It decreases with concrete member strength grade after it exceeds C65. Due to the member's damage, it can no longer successfully convert impact energy into internal energy. The right end support's energy absorption capacity changes with concrete strength; however, the left end support's energy absorption capacity improves with concrete strength. The concrete on the right side of the impact point fails, and the force imposed by the impact body on the members is transferred to both ends through the longitudinal steel bars.

**Table 7:** Related data statistics of different concrete strength members.

No	Peak (kN)	Left support (kN)	Delay (ms)	Right support (kN)	Delay (ms)	Maximum deflection (ms)	Energy absorbing (J)	Left energy absorption (J)	Right energy absorption (J)
CSG1	279.94	− 83.01	1.25	271.87	0.30	134.04	4903.43	1203.55	3645.02
CSG2	288.59	− 94.02	1.25	268.97	0.30	132.80	5043.10	1190.37	3758.58
CSG3	299.00	− 84.31	1.15	271.20	0.30	136.81	5027.54	1205.07	3730.17
CSG4	307.33	− 82.94	1.15	288.48	0.25	168.33	4967.03	1416.82	3460.99
CSG5	315.22	− 77.96	1.05	304.72	0.25	173.98	4896.60	1439.57	3345.43

The downward direction of the impact force is positive, and the upward direction of the reaction force is positive.

The partially completed concrete section carries it to the impact point's side. When the concrete strength is more than C65, the members are fractured at the impact point, and the left side of the members becomes a cantilever beam that continues to sustain the impact load.

In summation, concrete strength between C45 to C65 does not influence member impact resistance. However, when the concrete strength exceeds C65, it becomes brittle, reducing the members' impact resistance and making them easy to fracture.

### Effect of Impact velocity

**Figure 15:**
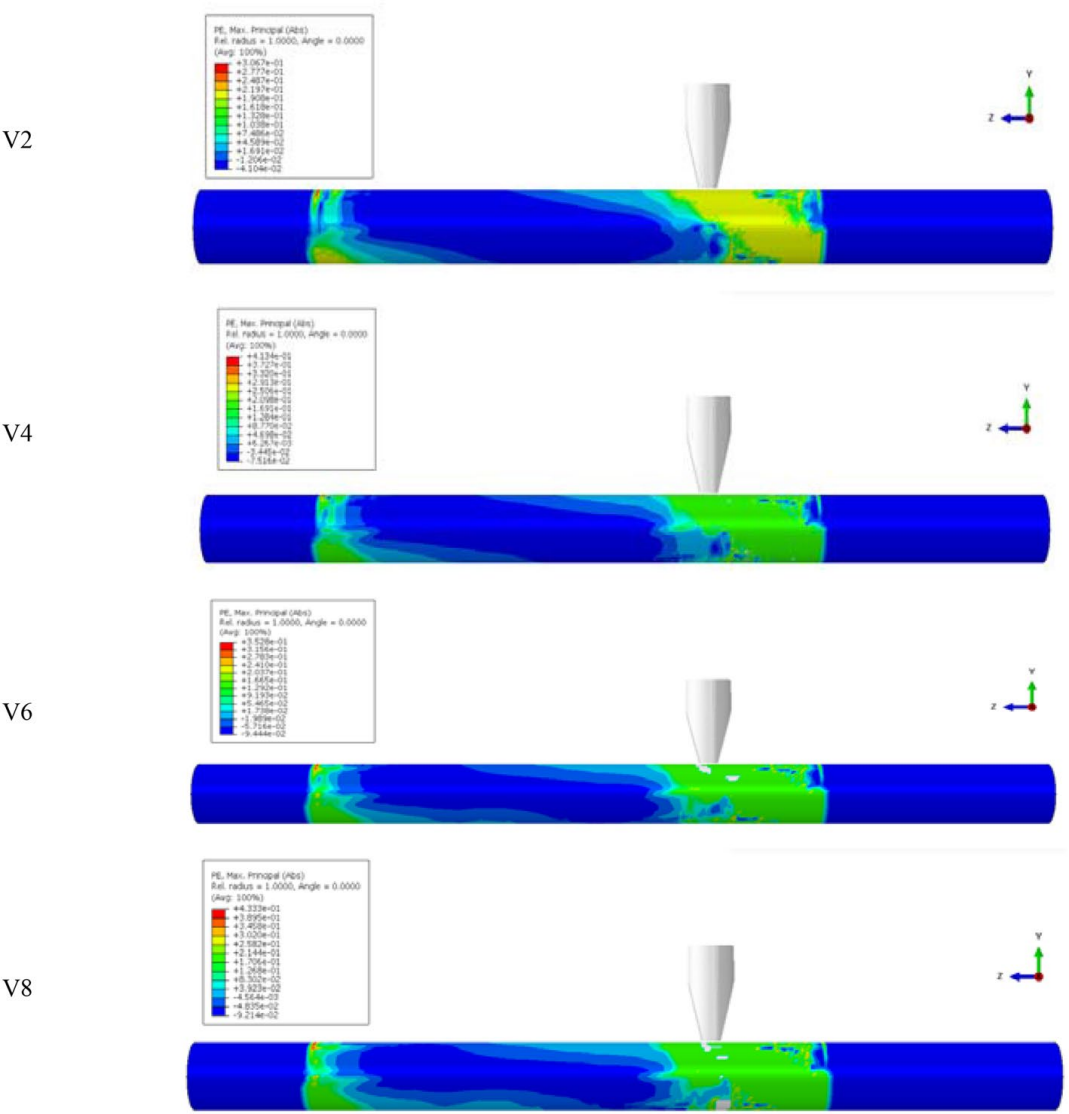
Plastic strain diagrams of members with different impact velocities.

In the experimental test results, the members have been extensively damaged by high-impact energy, although YH1 and YH2 have varying degrees of damage. Other parameters are held constant to evaluate the impact energy on the impact resistance of reinforced concrete members. The impact velocity changes the impact energy. Parameters V1, V2, V3, V4, V5, V6, V7, V8, V9, and V10, are computed for the impact height range of 2 m beginning from 0.2 m with a 0.2 m gradient. In Fig. 15, the plastic strain shows V2, V4, V6, and V8 stress waves transferred to the supports at both ends. The figure shows that when the impact velocity is just 2.80 and 3.96 m/s, there are only bending fractures in V2 and V4. No shear damage occurs when a bending fracture enters the cross-section. The residual deflection of the two members exceeds 1.1% of the clear span, indicating a bending failure. When the impact velocity is 4.85 m/s, shear fractures occur on the right side of the impact point, along with bending cracks. The two kinds of fractures converged around the impact point and penetrated practically simultaneously, causing bending and shear failure. The diagonal shear crack on the right side of the impact point penetrates before the bending crack at the bottom. The member suffers a shear failure. These behaviors indicate that when the impact velocity is 4.85 m/s (V6) and the impact energy is 3175 J, the member will bend and shear. At 5.60 m/s (V8), the impact energy is 4233 J, generating shear failure.

**Figure 16:**
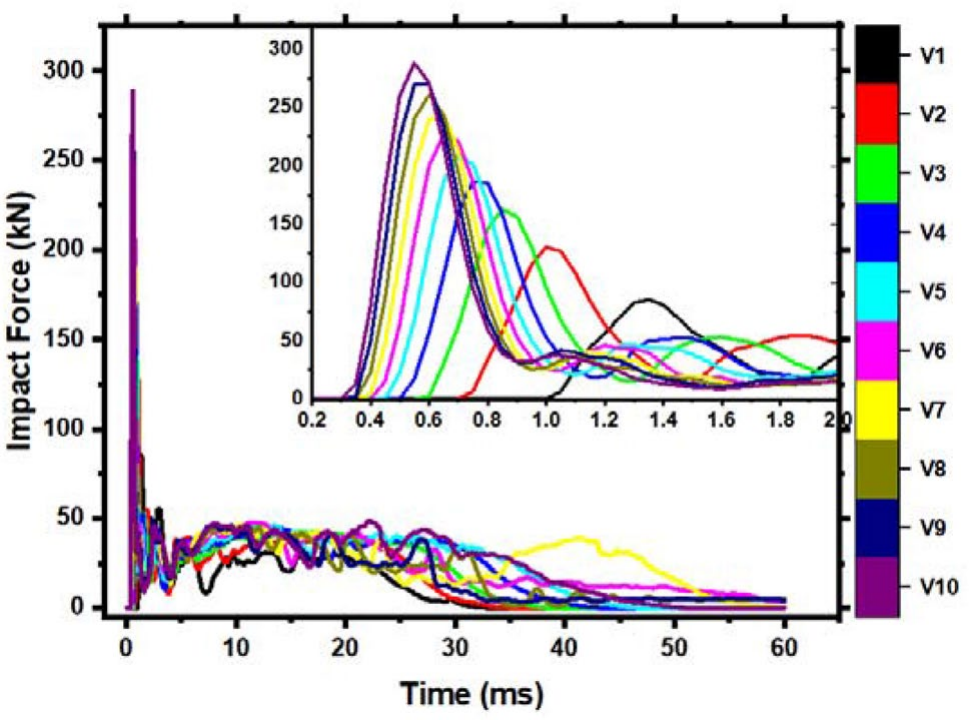
Impact force–time history curve of different impact velocity members.

Figure 16 compares the impact force–time histories of each member. The figure shows that when the impact velocity increases, the peak force increases, and the impact action time increases. The impact force fluctuation pattern of each component is the same. The increase in plateau force is not noticeable at 3.43 m/s impact velocity. The member section bearing capacity is maximum once the impact velocity is 3.43 m/s. Hence, the member can only depend on increased deformation to absorb impact energy.

**Figure 17:**
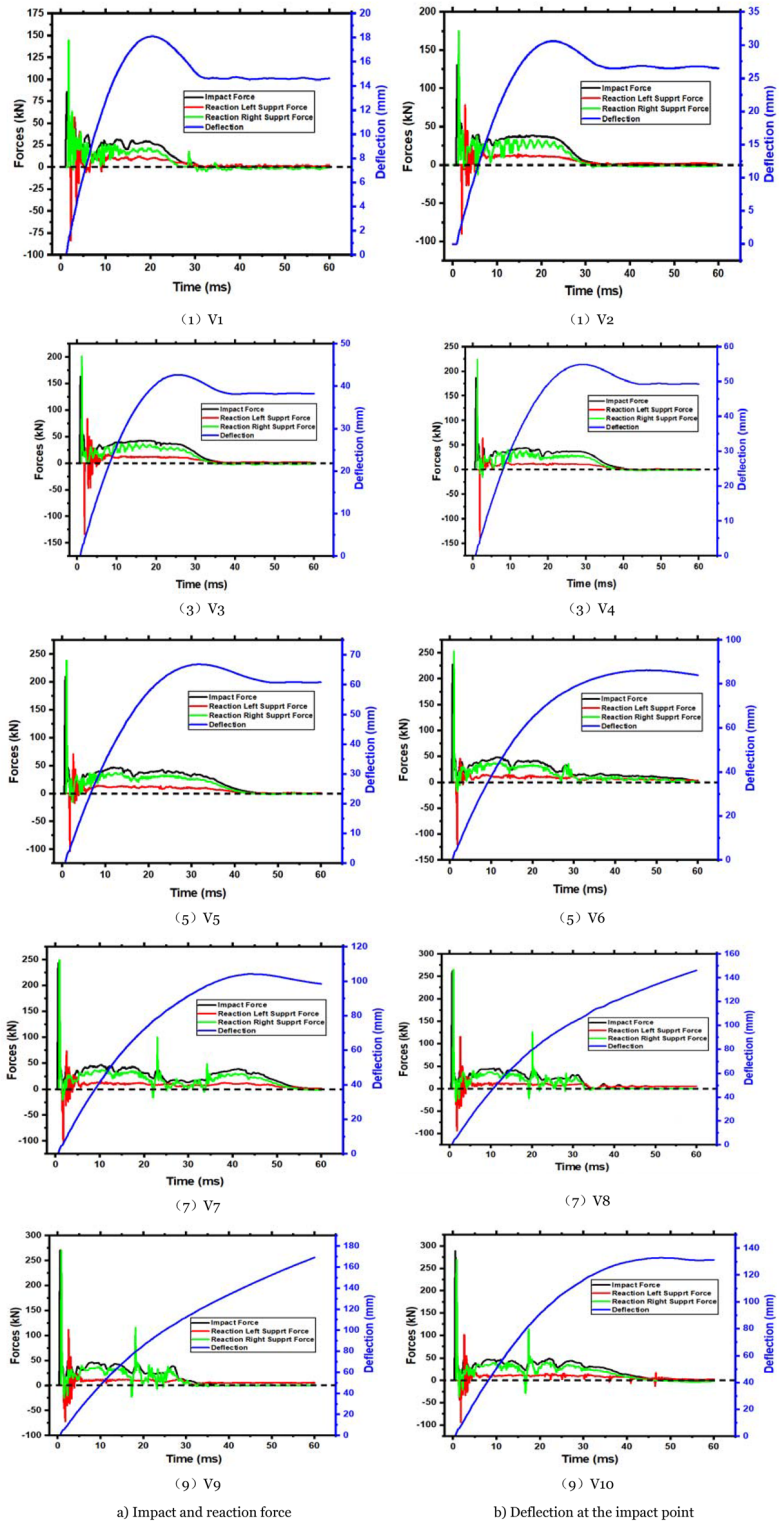
Forces and deflection time history curves of members under different impact velocities.

The member's force–time history curve at various impact velocities is shown in Fig. 17a. At the left end of V1, V2, and V3 increase with impact velocity before decreasing. This phenomenon is related to member failure. Less than half of the first four members bend, causing bending-shear failure. Due to local damage, the severe stress wave cannot be fully transmitted to the support as the impact velocity increases. The response force of the first four members has a longer variation time. The time of fluctuation is decreased as the impact speed increases. However, there is a dramatic rise and fall in the steady phase due to slow transmission and reflection time stress waves. In addition, the local fracture of the concrete on the right side of the impact point redistributes the cross-sectional stress, causing sudden peaks and troughs. Under varying impact velocities, a member's deflection time history curve is shown in Fig. 17b. The member deflection increases with impact velocity. Less than 5.24 m/s impact velocity causes rebound. At this point, the member is elastoplastic damaged. When the velocity exceeds 5.24 m/s, the deflection of the member increases. The member loses its bearing capacity and is difficult to maintain.

**Figure 18:**
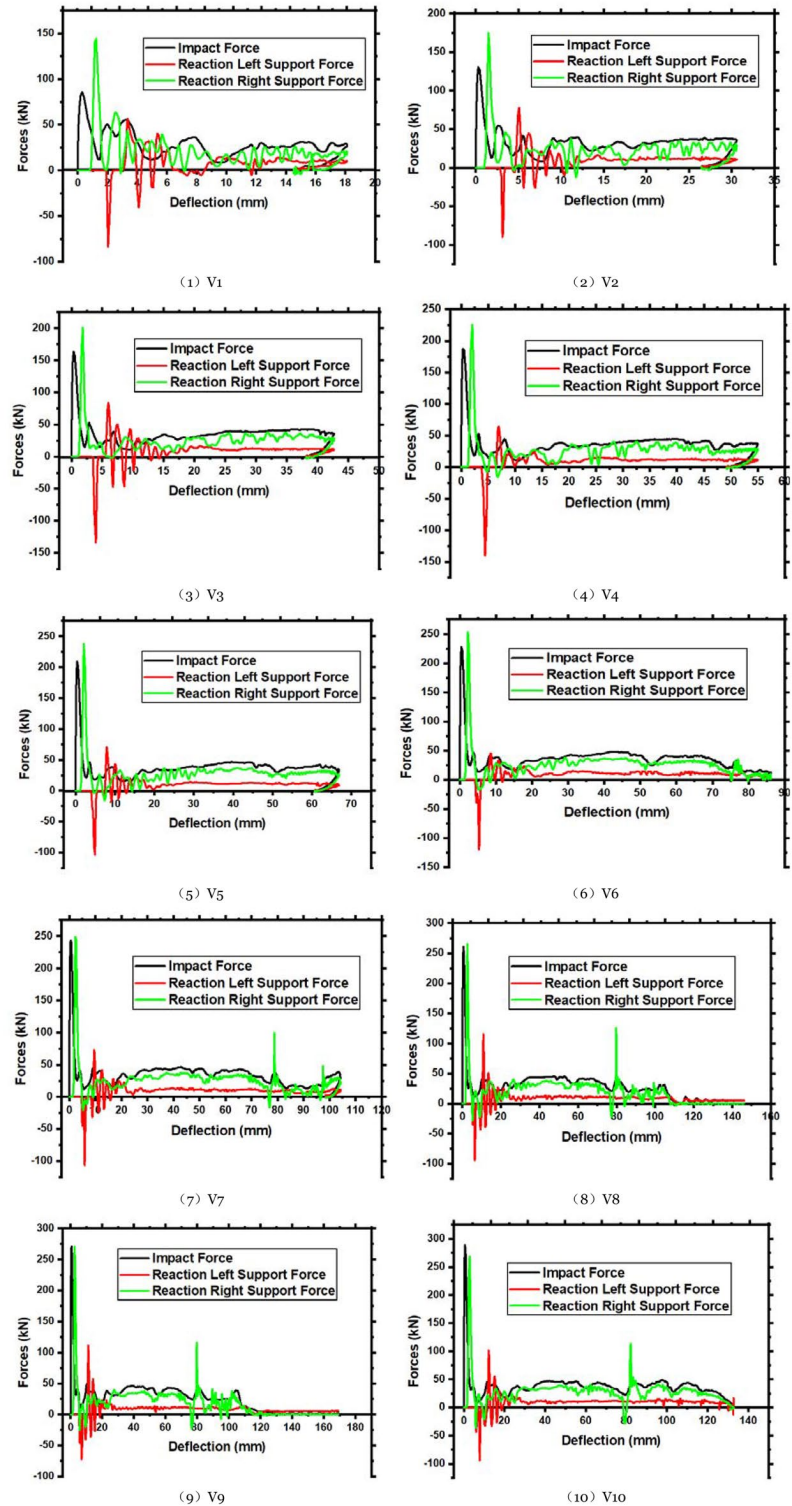
Force–deflection curve of the members under different impact velocities.

The member force–deflection curve is in Fig. 18. Integrate the curve into the table to determine the member's energy. Table 8 has precise data for various impact velocities. As the impact velocity increases, so does the member’s energy absorption. When bending occurs, the member's force–deflection curve resembles a parallelogram. This phenomenon has been studied extensively.

**Table 8:** Data analysis of relevant member’s results at various impact velocities.

Member no	Peak (kN)	Left support (kN)	Delay (ms)	Right support (kN)	Delay (ms)	Maximum deflection (ms)	Energy absorbing (J)	Left energy absorption (J)	Right energy absorption (J)
V1	85.50	− 83.74	0.90	144.33	0.40	18.10	488.92	113.57	360.15
V2	130.58	− 89.87	1.00	174.62	0.35	30.58	979.81	264.98	699.75
V3	163.39	− 134.10	1.05	201.26	0.35	42.67	1469.46	385.20	1057.78
V4	186.89	− 139.92	1.05	224.73	0.35	54.99	1973.20	487.63	1436.44
V5	209.33	− 103.52	1.10	238.18	0.30	66.87	2495.19	620.62	1836.94
V6	227.23	− 120.06	1.10	253.00	0.30	86.17	3079.57	790.60	2031.36
V7	243.61	− 106.27	1.10	249.10	0.25	104.23	3572.94	919.68	2578.99
V8	261.16	− 93.69	1.15	265.31	0.30	146.13	3977.51	1132.00	2727.06
V9	270.73	− 72.45	1.15	270.38	0.25	169.12	4301.33	1284.96	2940.96
V10	288.59	− 94.02	1.25	268.97	0.30	132.80	5043.10	1190.37	3758.58

The impact force is positive downward, while the reaction force is upward.

The impact velocity will generally influence the member's failure shape (i.e., impact energy). A velocity of impact less than 2.80 m/s causes bending failure; a velocity of impact between 2.80 m/s and 3.43 m/s causes critical bending and shear failure. Shear failure occurs when the impact velocity exceeds 3.96 m/s. The member may be entirely damaged if the impact velocity reaches 5.24 m/s.

### Effect of Stirrup ratio

**Figure 19:**
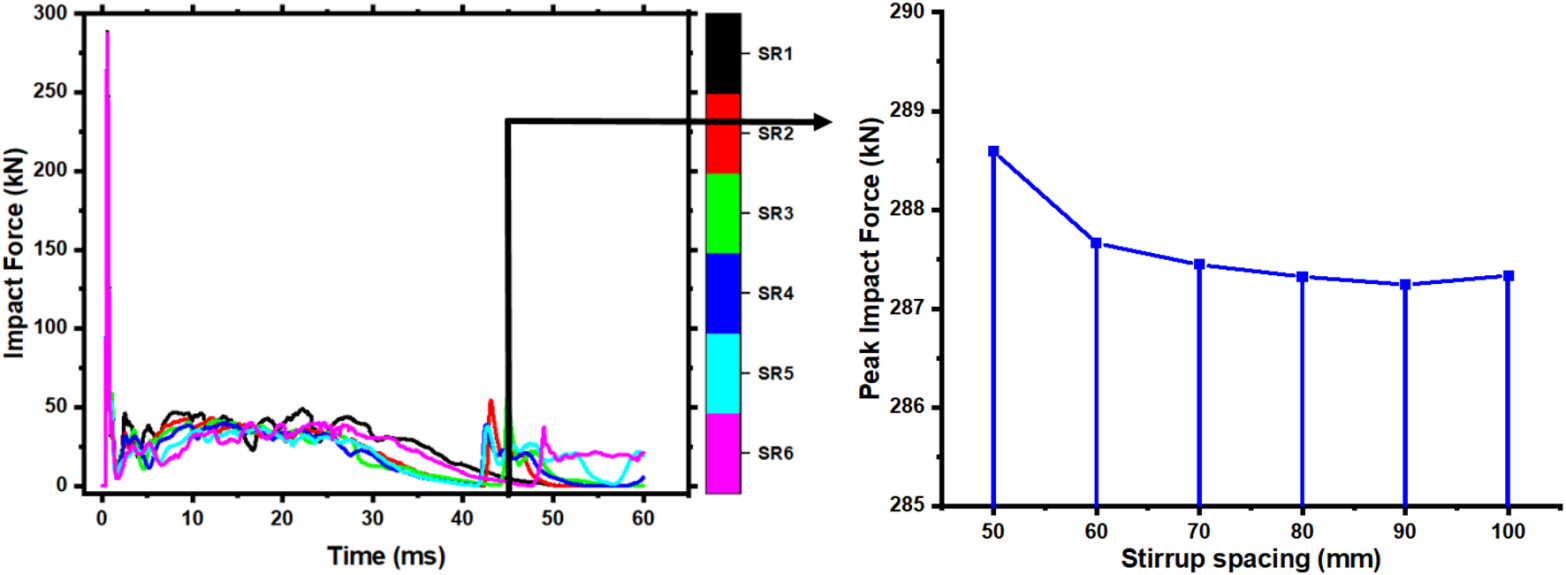
Peak and impact force–time history curve of members with various stirrup ratios.

**Figure 20:**
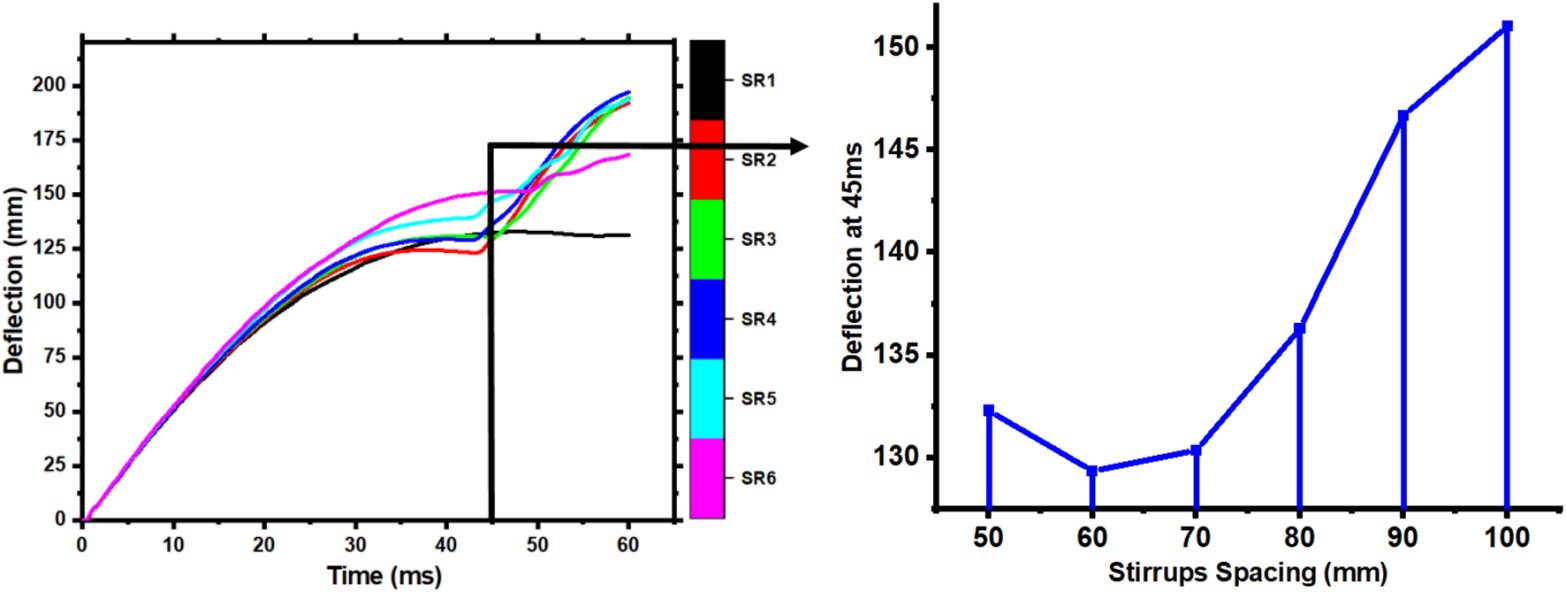
Deflection time history curve of members and comparison of deflection with various stirrup ratios at 45 ms.

Stirrups are utilized to meet the oblique section's shear strength and strengthen the member's integrity. To investigate the influence of the stirrup ratio on members' impact resistance, the stirrup spacing was altered as s = 60 mm, s = 70 mm, s = 80 mm, and s = 90 mm. At the same time, the other parameters remained the same. The condition s = 50 mm, and s = 100 mm investigates the members, and these parameters are denoted from small to wider spacing as SR1, SR2, SR3, SR4, SR5, and SR6. Figure 19 shows each member's impact force–time history curves and peak impact force. Change in stirrups ratio has little influence on impact force fluctuation trend and duration of the member, as seen by each curve in Fig. 19. The peak impact force reduces with stirrup spacing; however, it progressively decreases when stirrup spacing surpasses 70 mm. Compared to the deflection time history curves of various stirrup ratio members in Fig. 20. The figure reveals that each member's initial (15 ms) deflection variations are the same, indicating that the stirrups ratio does not affect the member's stiffness. After 15 ms, the deflection of the members increases with the stirrup spacing. Also, Fig. 20 illustrates each member's deflection at (45 ms). Deflection increased sharply after (45 ms) for practically all members with stirrup spacing > 50 mm. The increased spacing makes the stirrups more resistant to damage within the service range. It is difficult to sustain overall stressed condition after stirrups break, resulting in immediate fracture of members.

In conclusion, the stirrup ratio has little influence on the member's failure mechanism and impact force. Still, it may decrease the damage degree of the member and prevent it from being entirely broken.

### Effect of slenderness ratio

The slenderness ratio of the member may be computed by $\lambda = \frac{KL}{r}$ where (*L*) is the actual measured length of the member, (*K*) stands for effective length. The American Institute of Steel Construction (AISC) manual lists these values between 0.5 and 2.0^56^. The value of K depends on how the boundary condition members are attached in a structure (*K* = 0.5 for two fixed-end members), (r) is the circular section radius. Altering the member's diameter demands a lot of effort in the finite element, therefore, changing the member's length changes the slenderness ratio. The test scales its cross-sectional area, and its diameter is computed.

**Figure 21:**
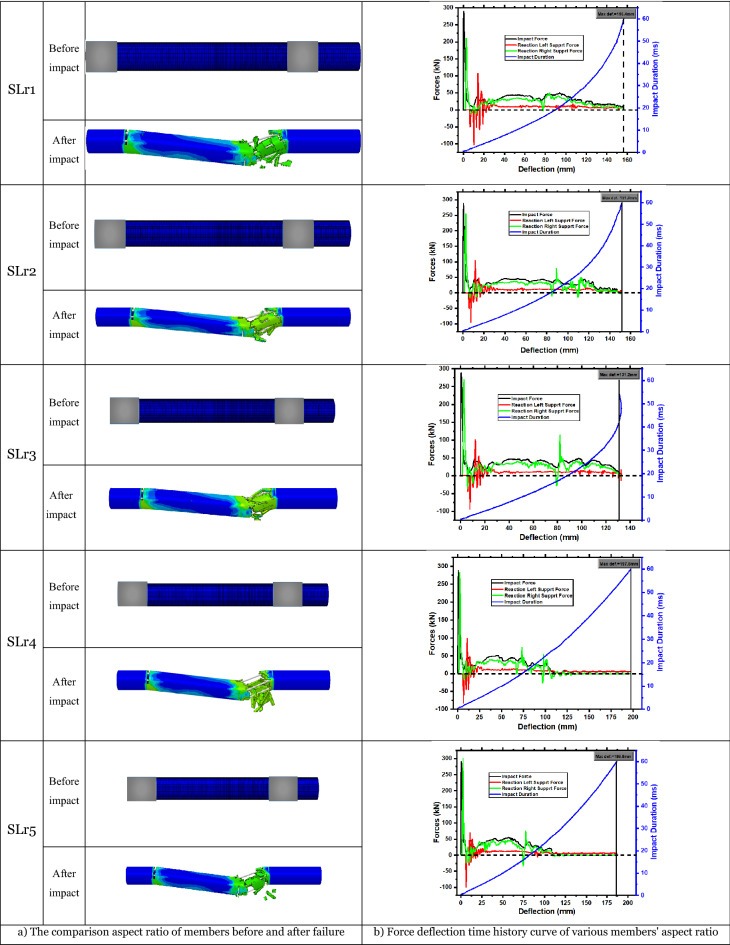
Effect of aspect ratio on the members.

The slenderness ratio is determined without affecting the member's length. Assume that the member's other criteria stay fixed and that the slenderness ratio alters the member's length. Each member's slenderness ratio for the specimen (YH2) is 19.41, 18.43, 17.55, 16.76, and 16.28. Their clear span lengths are 1006 mm, 950 mm, 900 mm, 855 mm, and 827 mm, respectively, with parameter names indicated in succession for members as SLr1, SLr2, SLr3, SLr4, and SLr5. Figure 21a depicts slenderness ratio comparisons before and after failure. The figure shows that all members' shear fails to the right of the impact point. In addition to the shear fracture, member SLr1 is bent and cracked at the bottom of the impact point, SLr2 and SLr3 are bent and cracked at the top of the right end support, and SLr4 and SLr5 are broken precisely where the shear crack emerges on the right side of the impact point.

The region of stress concentration grows as the member's slenderness ratio increases. The member's force–deflection time history curve is shown in Fig. 21b. The two members, SLr4 and SLr5, broke after being struck, and the member deflection increased. The remaining three members' deflection increased with the slenderness ratio. The slenderness ratio of SLr4 and SLr5 is small, the stress is focused on the right side, and the local stress is too high, leading the member to fracture. Deflection increases with the slenderness ratio for members that have not broken because deformability increases with member length.

Integrate the curve to absorb energy. The energy absorbed by the member and the right end support increases with the slenderness ratio. Still, the left end support absorbs less energy as the slenderness ratio increases. The member grows, impact resistance improves, and energy is consumed. The member may be bent and damaged as the slenderness ratio increases, allowing energy to be efficiently transmitted to the supports at both ends.

To summarize, the member's deformation ability, energy absorption capacity, and stress concentration area increase with the slenderness ratio. The peak value of the impact force reduces significantly, and the duration increases, but the impact force plateau remains the same. As the slenderness ratio increases, the member's failure mechanism changes from shear to bending. Once the slenderness ratio reaches 35.30, the member fails in bending-shear. Whenever the slenderness ratio exceeds 35.30, the member may fail by bending.

**Figure 22:**
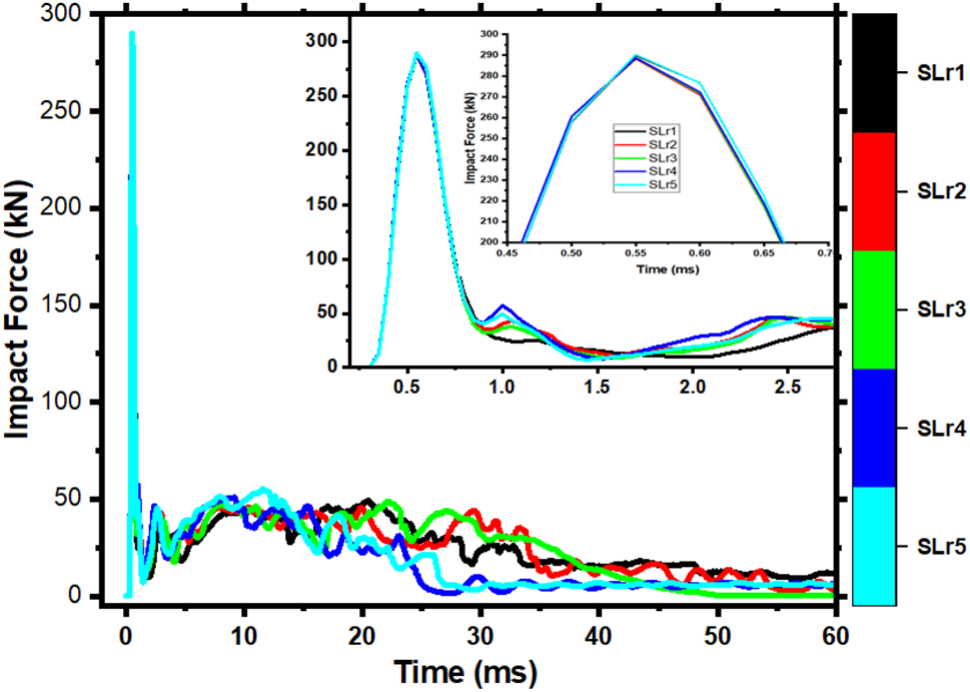
Impact force time-history curves with various aspect ratios of members.

Figure 22 compares the impact force–time history curves of the members, and the specific data for various aspect ratios are shown in Table 9. The impact duration increases and the peak impact force drops as the member's slenderness ratio increases, but this does not influence the plateau value.

**Table 9:** Statistics of members with various aspect ratios.

Members no	Peak (kN)	Maximum deflection (mm)	Impact energy (J)	Left energy absorption (J)	Right energy absorption (J)
SLr1	289.83	156.41	5128.76	1230.88	3711.72
SLr2	288.28	151.89	5131.49	1359.69	3673.82
SLr3	288.59	132.80	5043.10	1190.37	3758.58
SLr4	288.69	197.57	4528.05	1564.79	2847.96
SLr5	290.26	186.79	4667.22	1440.14	3042.35

## Conclusion

Under unequal span lateral impact loads, this paper focuses on five aspects of reinforced concrete members' dynamic response and failure mechanism. A drop hammer impact testing of reinforced concrete members exposed to unequal lateral impact. Establishing finite element models with the influence of various parameters. Unequal span high-velocity impact stresses cause shear failure. The right side of the impact point creates the shear failure surface. The top of the failing surface is the point of impact. Three-dimensional unequal span lateral impact finite element model of reinforced concrete members steel and concrete are included because of their strain rate impacts. Compare the test findings to the model's failure mechanism, impact force–time history curve, and deflection time curve simulation results. The results show that the finite element model proposed in this work accurately predicts reinforced concrete members forced mechanical characteristics and failure mechanisms. The findings of finite element analysis on the impact resistance reveal that:

1. Increasing a member's reinforcement ratio may help it withstand deformation. Conversely, over-reinforced above 6% of members fail early due to steel failure. Its energy absorption capacity decreases with the increase in the reinforcement ratio > 4.6%.
2. Concrete strength grades CSG1, CSG2, and CSG3 have minimal effect on member impact resistance. Concrete stronger than CSG3 (65 MPa) is easily fractured.
3. Members fail due to impact velocity (i.e., impact energy). If the impact velocity is less than 2.80 m/s, the member will deform. The member may bend and shear between 2.80 m/s and 3.43 m/s. Shear failure occurs at 3.96 m/s effects. At 5.24 m/s, the member may completely fracture.
4. The stirrups ratio does not affect the member failure mechanism or impact force. It may nevertheless decrease the damage and stop the member from becoming separated.
5. The slenderness ratio improves deformation, plateau value, energy absorption by ≤ 18.43% as well. The SLr1 element failed the bending test. With an increasing slenderness ratio, the peak impact force drops, and the member fails in bending rather than shear.

## Further research

It is essential to examine the specimens' response to axial force. Attempts should be made to identify feasible approaches to enhance the ability value associated with FRP sheet wrapping in reinforcement concrete. In the construction sector, the outcomes of such investigations would immediately assist.

## Data Availability

The datasets used and analysed during the current study are available from the corresponding author upon reasonable request.
